# Synthesis of Novel 1,4-Diketone Derivatives and Their Further Cyclization

**DOI:** 10.1021/acsomega.3c00610

**Published:** 2023-04-07

**Authors:** Hacer Can Üsküp, Tülay Yıldız, Hülya Ç. Onar, Belma Hasdemir

**Affiliations:** Department of Chemistry, Organic Chemistry Division, Istanbul University-Cerrahpaşa, Avcilar, Istanbul 34320, Turkey

## Abstract

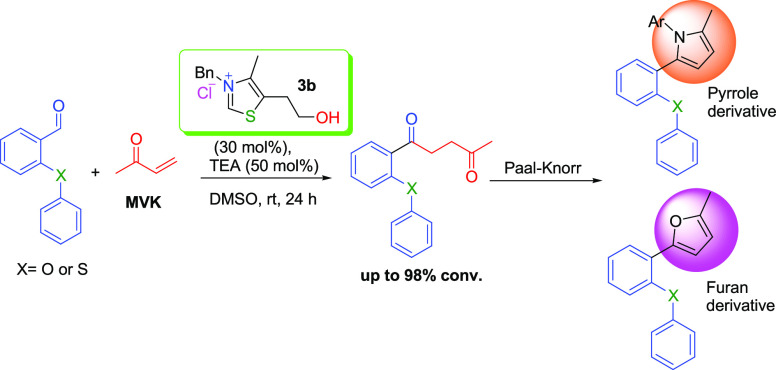

One of the important reactions to obtain a new carbon–carbon bond is the Stetter reaction, which is generally via a nucleophilic catalyst like cyanide or thiazolium-NHC catalysts. In particular, 1,4-diketones with very functional properties are obtained by the Stetter reaction with the intermolecular reaction of an aldehyde and an α,β-unsaturated ketone. In this study, we synthesized new derivatives (substituted arenoxy) of 1,4-diketone compounds (**2a**–**2n**) with useful features by a new version of the Stetter reaction method. In our work, arenoxy benzaldehyde derivatives with different structures as the Michael donor and methyl vinyl ketone as the Michael acceptor were used for the intermolecular Stetter reaction. The reaction was catalyzed by 3-benzyl-5-(2-hydroxyethyl)-4-methylthiazolium chloride (**3b**), using triethylamine for the basic medium and dimethyl sulfoxide as the solvent. As a result, some novel arenoxy-substituted 1,4-diketones were gained with good yields at room temperature within 24 h through an intermolecular Stetter reaction. In addition, new furan and pyrrole derivatives were prepared by performing the cyclization reaction with one of the obtained new diketone compounds.

## Introduction

The Stetter reaction is one of the significant carbon–carbon bond formation reactions using a nucleophilic catalyst. It has a different mechanism than the classical Michael addition,^1^ aldol reaction,^2,3^ and Mannich reaction,^4,5^ which makes other C–C bonds. It takes place by reaction of aldehydes with Michael acceptors of the 1,4 addition type with nucleophilic catalysts such as cyanide ions or *N*-heterocyclic carbenes (NHCs).^6^ First, aldehydes undergo the umpolung reaction along with a catalyst. To make the carbonyl carbon nucleophilic, the umpolung reaction allows carbon–carbon bond formation in milder conditions by reversing the actual polarity of the carbonyls.^7−14^ Then, a 1,4-addition reaction takes place with electrophilic carbon double bonds (Michael acceptors), and the creation of new carbon–carbon bonds takes place.^15−17^ This reaction was first discovered by Hermann Stetter in 1973 in the production of 1,4-dicarbonyl compounds and named after him.^18^ The reaction allows the synthesis of γ-keto nitriles, γ-keto esters, and γ-diketone products, which are important intermediates or starting materials in the synthesis of various heterocyclic molecules and bioactive heterocyclic systems found in natural products.^16,19,20^ It is very useful and versatile due to its applicability to substrates such as various heteroaromatic aldehydes and substituted aryl aldehydes. In the Stetter reaction, ketones, α,β-unsaturated esters, nitriles, aldehydes, and nitrous oxides are preferred as Michael’s receivers.^17^

Cyanide ion,^18,21^ thiazolium salt,^21,22^ bis(amino)cyclopropenylidenes,^23^ chiral bicyclic thiazolium salt,^20^ ThDP-linked enzymes like lyases, MenD, and PigD,^24,25^ and NHCs^9,13,16,26−32^ have been utilized as catalysts in the Stetter reaction. The first isolation of free carbenes was carried out independently by Bertrand et al.^33^ and Arduengo et al.,^34^ and this discovery led to the emergence of suitable approaches for obtaining medically and biologically important compounds.^9,30^ Recently, NHCs have performed effective reactions with homoenolates, enolates, vinyl enolates, acyl azoles, and acyl anion reagents to provide products that are not readily available by other means, and their interest in the field of catalytic synthesis is increasing.^35^ NHCs are excellent donors and form complexes containing strong metal–carbon bonds with thermal stability and higher catalytic activities. Simultaneously, singlet NHCs as unique Lewis bases are potent organocatalysts with both basicity and π acidity to allow the formation of a second nucleophile during a reaction (Breslow intermediate). As effective catalysts, NHCs are widely used in a variety of chemical syntheses and applications. Especially, the selective reactions mediated by chiral NHCs, high yield, and excellent regioselectivity, diastereoselectivity, or enantioselectivity aroused great interest.

**Scheme 1:**
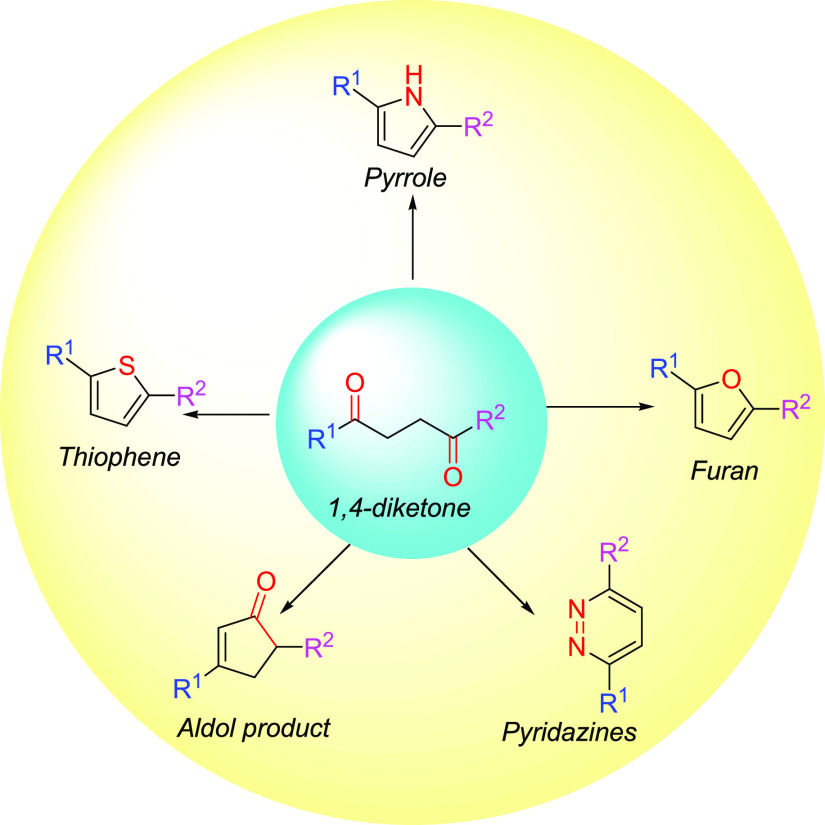
Derivatization of 1,4-Diketones

1,4-Diketones, with two carbonyl groups in one molecule, are an important structure frequently found in biologically active natural products.^36−38^ Additionally, they are very useful in the synthesis of some important heterocycles such as furan, thiophene, pyrrole, and pyridazines using Paal–Knorr synthesis^39−41^ (Scheme 1). These heterocycles are valuable building blocks of natural and pharmaceutical substances such as lophotoxin, non-natural amino acid Fmoc-d-3-Ala(2-thienyl)-OH, minaprine, and Lipitor.^42^ Synthesis of 1,4-dicarbonyl compounds is accomplished by one of the oxidative cross-coupling, nucleophile–electrophile coupling, or Stetter reactions.^43^ Synthesis of 1,4-diketones is more difficult than other 1,4-carbonyls, and they are obtained by coupling reactions of multifunctional substrates. In these coupling reactions, either multiple coupling partners are used or multiple pre-steps are required for the polyfunctionalization of a single partner.^44−48^ Especially, *ortho*-substituted alkoxy or arenoxy groups (salicylaldehyde derivatives) have some very important biological activities, such as EP1 receptor antagonists.^49,50^ Also, there is a lack of this kind of original diketones and their furan and pyrrole derivatives in the literature. Since steric hindrance is more likely in *ortho*-substituted structures, it is our first priority to obtain these structures in high yields in this study.

**Scheme 2:**
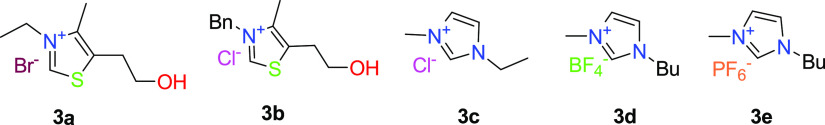
NHC Catalysts Used in the Stetter Reaction

Here, it was developed an optimized procedure of the Stetter reaction using some *ortho*-(thio)arenoxy benzaldehyde compounds (**1a**–**1n**) synthesized by us^51−53^ and methyl vinyl ketone. We tried some NHC catalysts (Scheme 2) in order to obtain a series of original *ortho*-(thio)arenoxy-substituted 1,4-diketone compounds (**2a**–**2n**), and 3-benzyl-5-(2-hydroxyethyl)-4-methylthiazolium chloride (**3b**) was found as the best NHC catalyst. The **3b** catalyst has been used in a previous Stetter reaction study for aliphatic aldehydes.^21^ For *ortho*-substituted benzaldehydes, ethyl-5-(2-hydroxyethyl)-4-methylthiazolium bromide (**3a**) was used and low–middle yields were obtained in the same study. As for our work, we used a **3b** catalyst for the first time to convert sterically hindered *ortho*-substituted arenoxy substrates to diketone derivatives under mild conditions and obtained high yields. In addition, new furan (**4a**) and pyrrole derivatives (**4b** and **4c**) were synthesized by cyclization using one of the synthesized 1,4-diketones (**2g**) by the Paal–Knorr reaction.

## Results and Discussion

The phenoxy or thiophenoxy benzaldehydes (**1a**–**1n**), our starting materials, were obtained by a substitution reaction of 2-fluorobenzaldehyde and substituted (thio)phenols in the presence of K_2_CO_3_ in DMF at 175 °C. The optimization study of the reaction of *o*-(thio)phenoxy benzaldehydes with methyl vinyl ketone (MVK) began by performing the conditions previously indicated for another Stetter reaction of salicylaldehyde derivatives.^49^ Then, we tested various NHC catalysts (**3a**–**3d**) and KCN that might support the Stetter reaction in order to obtain 1,4-diketone derivatives of corresponding *o*-(thio)phenoxy benzaldehydes and MVK. The NHC catalysts used in our experiments and whose formulas are given in Scheme 1 are as follows: 3-ethyl-5-(2-hydroxyethyl)-4-methylthiazolium bromide (**3a**), 3-benzyl-5-(2-hydroxyethyl)-4-methylthiazolium chloride (**3b**), 1-ethyl-3-methylimidazolium chloride (**3c**), 1-butyl-3-methylimidazolium tetrafluoroborate (**3d**), and 1-butyl-3-methylimidazolium hexafluorophosphate (**3e**). The results of our catalyst screening studies are summarized in Table 1.

**Table 1:**
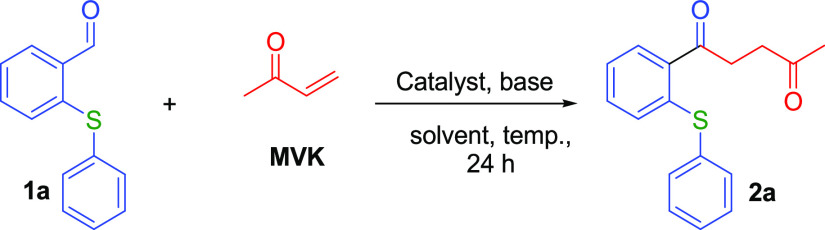
Catalyst Trials in the Stetter Reaction of **1a**^a^

entry	cata. (10 mol %)	base (20 mol %)	temp. (°C)	solvent		conv. (%)^b^
1	KCN		35	DMF		0
2	KCN	TEA	100	DMF		0
3	KCN	NaOH	70	EtOH		0
4	**3a**	TEA	70	EtOH		2
5	**3a**	TEA	100	DMF		0
6	**3a**	TEA	80	*i*-PrOH		5
7	**3a**	TEA	80	*t*-BuOH		8
8	**3a**	TEA	100	DMSO		40
9	**3a**	TEA	100	DMSO:H_2_O (8:2)		29
10	**3a**	TEA	100	THF		0
11	**3a**	TEA	25	DMSO		45
**12**	**3b**	**TEA**	**25**	**DMSO**		**80**
13	**3c**	TEA	25	DMSO		5
14	**3d**	TEA	25	DMSO		0
15	**3e**	TEA	25	DMSO		0

a Conditions: **1a** (0.1 mmol), MVK (2.5 mmol), catalyst (10 mol %), and base (20 mol %) in solvent (1 mL) were mixed at the stated temperature for 24 h.

b Conversions were identified with GC–MS.

As can be seen from Table 1, KCN was the first used catalyst with different bases and solvents. But it was found that KCN was ineffective to obtain a 1,4-diketone product of **1a**. Then, further experiments were made with **3a** from NHC catalysts. Then, we tried the most commonly used solvents and bases in similar reactions. For solvent trials, triethylamine (TEA) as a base and various organic polar protic (EtOH, *i*-PrOH, and *t*-BuOH) and aprotic solvents (DMF and DMSO) and also a nonpolar solvent (THF) were used in these experiments. Interestingly, among the solvents used, DMSO was the only one that showed good results. DMSO was used both at 100 °C (entry 8) and at room temperature (entry 11), and better conversion was obtained at room temperature in the presence of the **3a** catalyst. For this reason, DMSO was preferred as a solvent in the investigation of the other NHC catalysts (entries 12–15) at room temperature. It was observed that thiazolium catalysts and especially 3-benzyl-5-(2-hydroxyethyl)-4-methylthiazolium chloride (**3b**) gave the best result (entry 12), while imidazolium derivatives (**3d** and **3e**) had no activity among NHC catalysts (entries 14 and 15).

In our subsequent experiments, we used the **3b** catalyst and tested different bases and catalyst ratios to further increase the yield (Table 2). DBU, DMAP, KO*t*Bu, imidazole, and benzimidazole were used as bases in these experiments. However, it was determined that other bases did not show higher conversion than TEA. When we changed the amount of the catalyst and bases, it was seen that the highest conversion was obtained when a 30% equivalent **3b** catalyst and 50 mol % TEA were used (entry 10). When the reaction time was also controlled, it was observed that the highest result was reached in 24 h with 98% conversion.

**Table 2:**
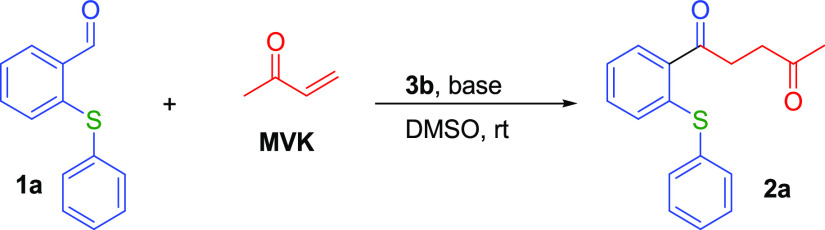
Exploration of Bases for the Stetter Reaction of **1a**^a^

entry	cat. amount (mol %)	base (mol %)	time (h)	conv.^b^
1	10	TEA (20)	24 h	80
2	10	DBU (20)	24 h	0
3	10	DMAP (20)	24 h	0
4	10	KO*t*Bu (20)	24 h	15
5	10	imidazole (20)	24 h	0
6	10	benzimidazole (20)	24 h	0
7	5	TEA (20)	24 h	75
8	20	TEA (20)	24 h	85
9	30	TEA (20)	24 h	90
**10**	**30**	**TEA (50)**	**24 h**	**98**
11	30	TEA (50)	12 h	92
12	30	TEA (10)	24 h	80

a Conditions: **1a** (0.1 mmol), MVK (2.5 mmol), catalyst **3b**, and base in solvent (1 mL) were mixed at room temperature for 24 h.

b Conversions were identified with GC–MS.

By using the new optimized Stetter method, condensation products of phenoxy aldehydes (**1a**–**1n**) prepared by using different substituted phenols and thiophenols with MVK were obtained. Thus, the method has been shown to be effective in a wide range of products. Looking at the isolated yields of the newly synthesized 1,4-diketones, good results were obtained between 71 and 96% (Table 3).

**Table 3:**
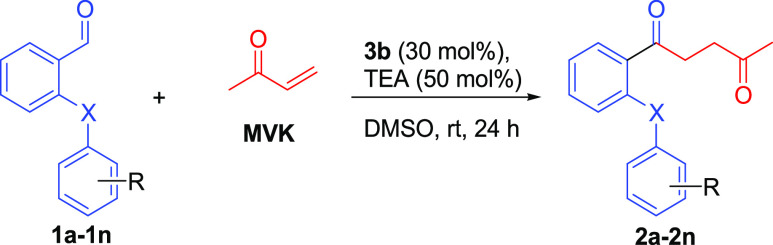

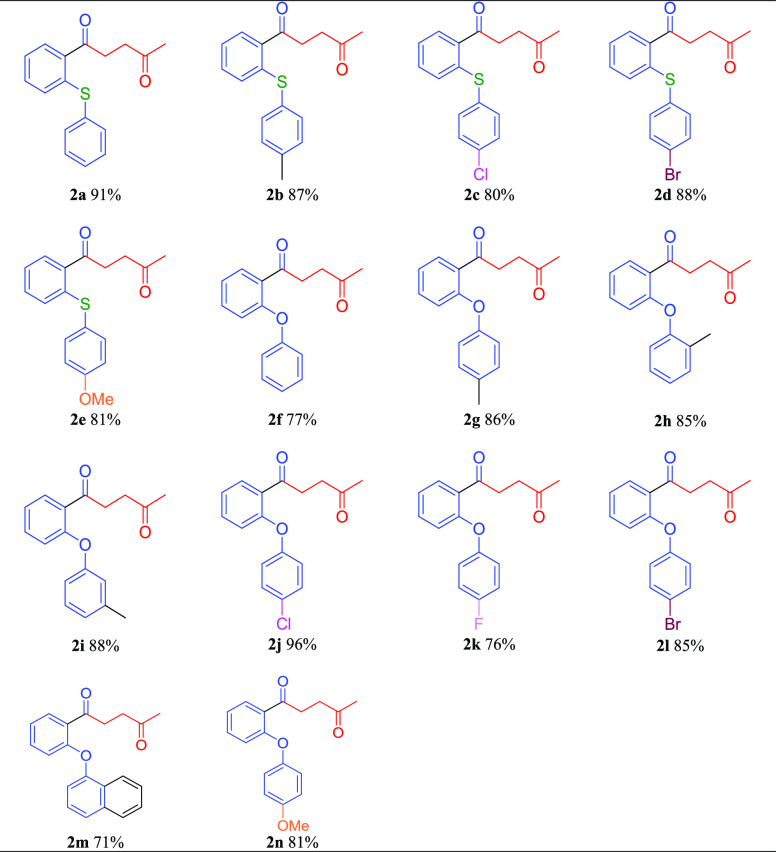
Scope of Substrates for the Stetter Reaction and Isolated Yields of the New 1,4-Diketone Products (**2a**–**2n**)^a^^,^^b^

a Conditions: **1a**–**1n** (0.1 mmol), MVK (2.5 mmol), catalyst **3b** (30 mol %), and TEA (50 mol %) in DMSO (1 mL) were mixed at room temperature for 24 h.

b Yield of isolated product.

**Scheme 3:**
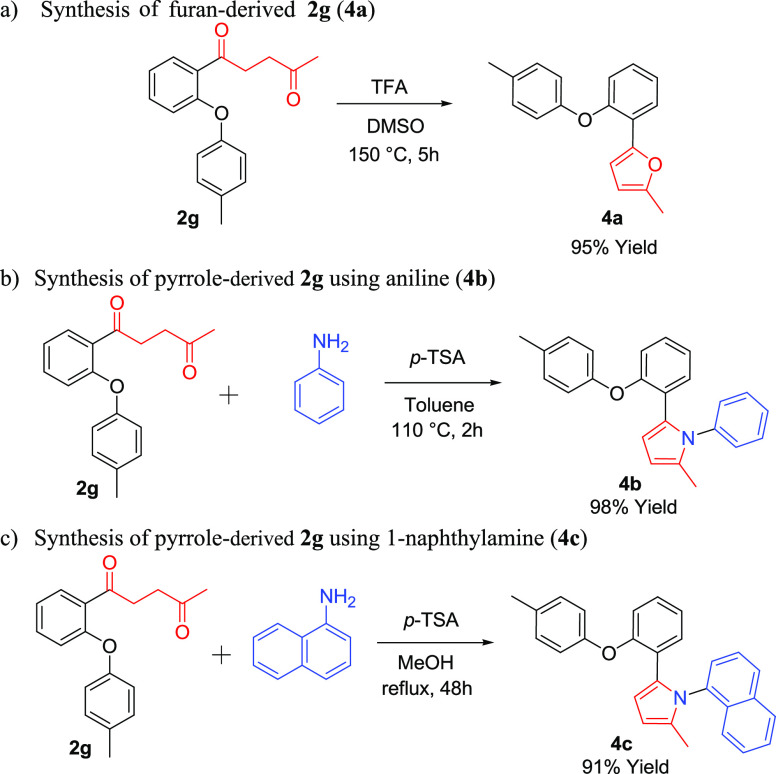
Derivatizations of 1,4-Diketone Compound **2g**

We wanted to show that new derivatives of phenoxy- and thiophenoxy-derived 1,4-diketones in the original structure can be obtained by cyclization reactions. For this reason, we carried out a series of derivatization studies. In these studies, we performed the **2g** diketone compound. We used different reagents for the cyclization experiments. First, we performed its reaction with trifluoroacetic acid in DMSO at 150 °C. After 5 h, it was observed that the desired furan derivative (**4a**) product was formed in 95% yield (Scheme 3a).

Our second derivatization reaction is the synthesis of pyrrole-derived **2g** using aniline (**4b**). In this reaction, a 98% yield was obtained using *p*-toluene sulfonic acid (*p*-TSA) in toluene at 110 °C and 2 h (Scheme 3b). We obtained a new pyrrole derivative using 1-naphthylamine as the third derivatization reaction (**4c**). In this experiment, a 91% yield was observed after the diketone and amine refluxing in MeOH for 48 h (Scheme 3c).

## Conclusions

In this study, the new 1,4-diketone products obtained as a result of the NHC-catalyzed Stetter reaction are important intermediates that can be used in drug synthesis, thanks to their molecular structures. Apart from that, it can form a starting material or intermediate product in many different organic syntheses. In particular, the structures synthesized in this study are very suitable for the synthesis of new heterocyclic compounds, which will increase the biological activity by the Paal–Knorr synthesis. Therefore, we were able to obtain three new heterocyclic derivatives by cyclization reaction using one of the new 1,4-diketones. With the synthesis of such compounds, it will be possible to discover new compounds with high biological activity. In particular, it has been shown that similar structures with *ortho*-substituted alkoxy or arenoxy groups increase their biological activities.^49,50^ Finally, we succeeded in developing a method for the synthesis of arenoxy-derived 1,4-diketones in original structures, which can be the precursors of new heterocyclic compounds.

## Experimental Section

### General Information

The predominance of the materials used in this work was commercially available from Acros, Merck, and Aldrich. The starting compounds **1a**–**1n** were prepared by a reaction of 2-fluorobenzaldehyde and substituted phenol or thiophenol compounds. The whole new products were described by IR, ^1^H-NMR, ^13^C-NMR, GC–MS, and elemental analysis. The reactions were observed using TLC by silica gel plates and the products were made pure by column chromatography systems on silica gel (Merck; 230–400 mesh), eluting with hexane–ethyl acetate (v/v 9:1). GC–MS were recorded on a Shimadzu QP2010 Plus. The IR spectra were recorded on a Mattson 1000 spectrometer. The NMR spectra were recorded at 500 or 400 MHz for ^1^H and 125 or 101 MHz for ^13^C using Me_4_Si as the internal standard in CDCl_3_. Melting points were measured using Büchi Melting Point B-540.

### General Procedure for the Stetter Reaction to Synthesize 1,4-Diketones

To a solution of a starting aldehyde compound (**1a**–**1n**) (0.1 mmol), MVK (2.5 mmol), catalyst **3b** (30 mol %), and TEA (50 mol %) in DMSO (1 mL) were mixed at room temperature for 24 h. After completion of the reaction as monitored on TLC, the solution was concentrated in vacuo and was extracted with DCM. Then, the common reaction workup and concentration were done, and the remaining product was purified by column chromatography with a mixture of hexane and ethyl acetate (v/v 9:1).

## Supporting Information Available

Experimental details, general procedures, experimental characterization data (IR, NMR, MS, and elemental analysis), and ^1^H and ^13^C nuclear magnetic resonance spectra of all products (PDF)
