# Supplementary material for: Synthesis of Novel 1,4-Diketone Derivatives and Their Further Cyclization
Source: ACS Omega. 2023 Apr 7;8(15):14047–52. doi: 10.1021/acsomega.3c00610 (PMC10116510; doi:10.1021/acsomega.3c00610)
Supplement: Supplementary file 1 — ao3c00610_si_001.pdf [file ao3c00610_si_001.pdf]

Supporting Information for

The synthesis of novel 1,4-diketone derivatives and their further cyclization

Hacer Can, Tülay Yıldız,* Hülya Ç. Onar and Belma Hasdemir

Istanbul University-Cerrahpaşa, Department of Chemistry, Organic Chemistry Division, Avcilar, Istanbul, 34320, Turkey
e-mail: tulayyil@iuc.edu.tr

Content

General information……………………………………………………………………S2

General procedure for the coupling reaction ………………………………………S2

General procedure for the Stetter reaction…………………………………………S2

General procedure for the cyclization reaction of diketone 2g ………………..S3

Experimental Characterization Data of 2a-2n and 4a-4c………………………..S4

NMR Spectra of all the reported compounds……………………………………S10

1. General Information

The majority of the chemicals used in this work were commercially available from Merck or Aldrich. The starting *ortho*-(thio)arenoxy benzaldehyde compounds (**1a-1n**) were prepared by coupling reaction of 2-fluoro benzaldehyde and substituted phenols or thiophenols All substrates were purified by crystallization or column chromatography and were characterized by IR, and GC-MS. All novel products were characterized by IR, ^{1}H-NMR, ^{13}C-NMR, elemental analysis, and GC-MS. The reactions were monitored by TLC using silica gel plates and the products were purified by flash column chromatography on silica gel (Merck; 230–400 mesh), eluting with hexane-ethyl acetate (v/v 9:1). NMR spectra were recorded at 500 MHz for ^{1}H and 125 MHz for ^{13}C using Me_4Si as the internal standard in $CDCl_3$. GC-MS were recorded on Shimadzu/ QP2010 Plus. IR spectra were recorded on a Bruker Vertex 70 IR spectrometer.

2. General procedure for the coupling reaction:

To a solution of DMF (10 mL) containing 2- fluoro benzaldehyde (5.0 mmol) and phenol or (thio)phenol (5.0 mmol) was added K_2CO_3 (5.0 mmol) and the reaction mixture was stirred for 2 hours at 180 ^{0}C under a nitrogen atmosphere. It was cooled to room temperature and after the usual workup and concentration, the product was purified over silica gel. Thus, *ortho*-(thio)arenoxy benzaldehyde was prepared in good yields with 95-100% (Scheme 1).

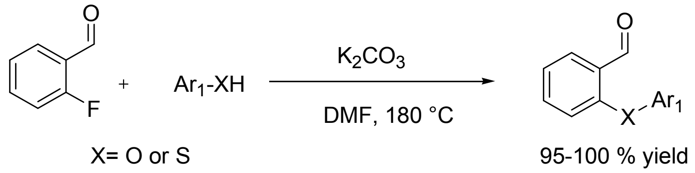

Scheme S1. The Ullmann-type reaction of 2-fluoro benzaldehyde and (thio)phenols.[1]

[1]: Reprinted (Adapted or Reprinted in part) with permission from [Yildiz, T.; Küçük, H. B. An Organocatalytic Method for the Synthesis of Some Novel Xanthene Derivatives by the Intramolecular Friedel-Crafts Reaction. RSC Adv. 2017, 7 (27), 16644–16649.]. Copyright [2017/Tülay Yıldız] [Royal Society of Chemistry/Tülay Yıldız].

3. General procedure for the Stetter reaction:

To a stirred solution of *ortho*-(thio)arenoxy benzaldehydes (**1a-1n**) (0.1 mmol), MVK (2.5 mmol), catalyst **3b** (30 mol%), and TEA (50 mol%) in DMSO (1 mL) were stirred at room temperature for 24 hours. After the completion of the reaction as observed on TLC, the mixture was concentrated in vacuo and was extracted with CH_2Cl_2. After the usual reaction

workup and concentration, the remaining product is purified by column chromatography with a mixture of hexane and ethyl acetate (v/v 9:1).

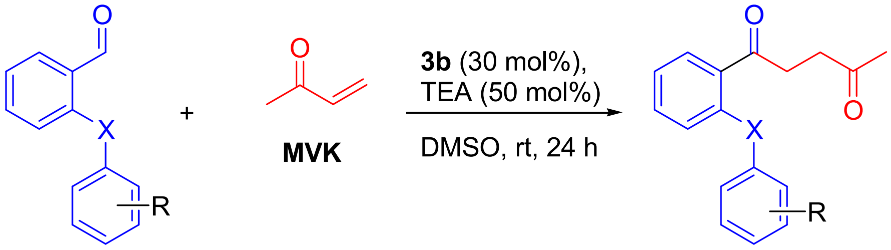

1a-1n

2a-2n

Scheme S2. The Stetter reaction of *ortho*-(thio)arenoxy benzaldehydes.

4. General procedure for the cyclization reaction of diketone 2g:

a) To a stirred solution of **2g** diketone with trifluoroacetic acid (TFA) in DMSO were stirred at 150 °C for 5 hours. After the completion of the reaction as observed on TLC, the mixture was concentrated in vacuo and was extracted with CH_2Cl_2. After the usual reaction workup and concentration, the remaining product is purified by column chromatography with a mixture of hexane and ethyl acetate (v/v 9:1).

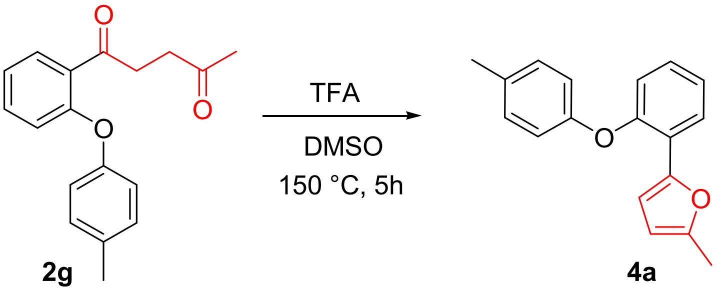

2g

4a

95% Yield

Scheme S3. The furan derivative of diketone **2g**.

b) To a stirred solution of **2g** diketone (0.1 mmol) and aniline (0.1 mmol) with *p*-toluene sulfonic acid (*p*-TSA) (10 %mol) in toluene were stirred at 110 °C for 2 hours. After the completion of the reaction as observed on TLC, the mixture was concentrated in vacuo and was extracted with CH_2Cl_2. After the usual reaction workup and concentration, the remaining product is purified by column chromatography with a mixture of hexane and ethyl acetate (v/v 9:1).

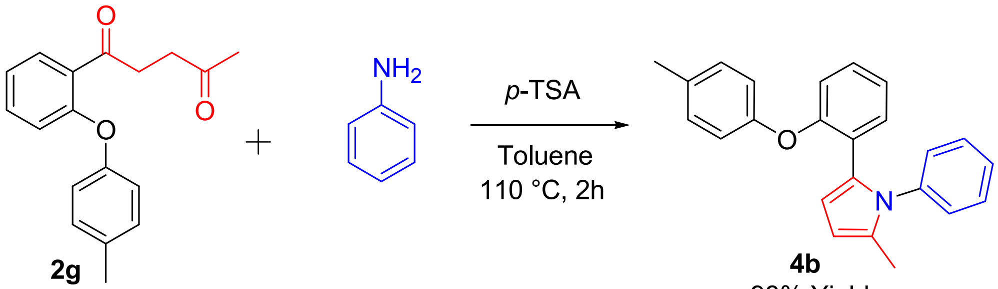

2g

4b

98% Yield

Scheme S4. The pyrrole derivative of diketone **2g** using aniline.

c) To a stirred solution of **2g** diketone (0.1 mmol) and 1-naphthylamine (0.1 mmol) with *p*-toluene sulfonic acid (*p*-TSA) (10 %mol) in methanol were refluxed for 48 hours. After the completion of the reaction as observed on TLC, the mixture was concentrated in vacuo and was extracted with CH_2Cl_2. After the usual reaction workup and concentration, the remaining product is purified by column chromatography with a mixture of hexane and ethyl acetate (v/v 9:1).

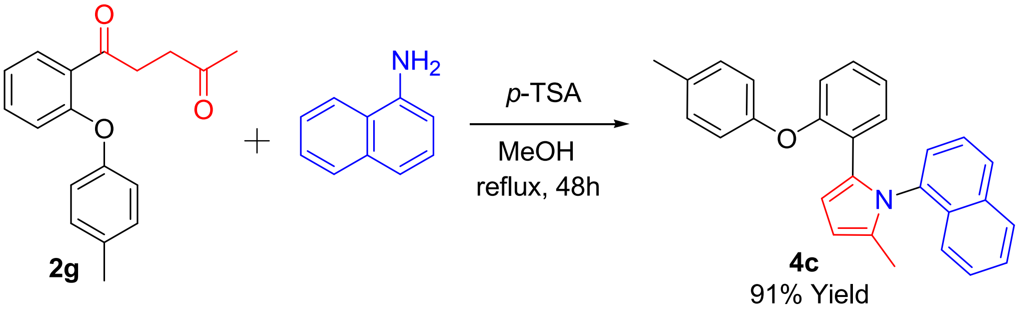

Scheme S5. The pyrrole derivative of diketone **2g** using1-naphthylamine.

5. Experimental Characterization Data of 2a-2n and 4a-4c

1-(2-(phenylthio)phenyl)pentane-1,4-dione (2a):

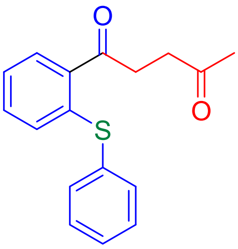

Pale orange oily. **IR** (cm^{-1}) ν3055, 2914, 2850, 1707, 1666, 1579, 1558, 1458, 1433, 1353, 1205, 1162, 1023, 990, 959, 739, 691. **^{1}H NMR** (500 MHz, $CDCl_3$) δ 2.29 (s, 3H), 2.92   2.96 (t, 2H, *J*=5.4 Hz), 3.30   3.33 (t, 2H, *J*=6.6 Hz), 6.89   6.91 (d, 1H, *J*=8.0 Hz), 7.18   7.22 (m, 1H), 7.28   7.32 (m, 2H), 7.42   7.43 (m, 2H), 7.53   7.54 (m, 2H), 7.93   7.95 (d, 1H, *J*=7.8 Hz). **^{13}C NMR** (150 MHz, $CDCl_3$) δ 30.1, 33.9, 37.2, 124.5   141.3 (12C), 199.6, 207.4. **MS** (m/z) = 43, 51, 77, 105, 131, 152, 184, 197, 213, 284 (M^+). Anal. Calcd. for $C_{17}H_{16}O_2S$: C, 71.80; H, 5.67; S, 11.28. Found: C, 71.76; H, 5.63, S, 11.15.

1-(2-p-Tolylsulfanyl-phenyl)-pentane-1,4-dione (2b):

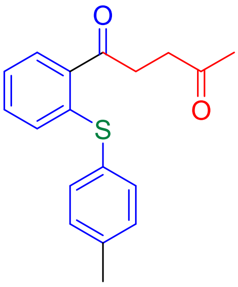

Orange oily. **IR** (cm^{-1}) ν 3075, 3019, 2912, 1712, 1671, 1584, 1556, 1492, 1464, 1433, 1394, 1353, 1269, 1233, 1203, 1159, 1139, 1044, 990, 957, 803, 757. **^{1}H NMR** (500 MHz, $CDCl_3$) δ 2.29 (s, 3H), 2.41 (s, 3H), 2.93   2.95 (t, 2H, J=6.2 Hz), 3.30   3.33 (t, 2H, J=7.3 Hz), 6.88   6.90 (d, 1H, J=8.1 Hz), 7.16   7.27 (m, 4H), 7.42   7.44 (m, 2H), 7.94   7.92 (d, 1H, J=7.8 Hz). **^{13}C NMR** (150 MHz, $CDCl_3$) δ 21.3, 30.1, 33.8, 37.2, 124.2   142.6 (12C), 199.5, 207.3. **MS** (m/z) = 43, 77, 91, 108, 139, 163, 184, 213, 227, 298 (M^+). Anal. Calcd. for $C_{18}H_{18}O_2S$: C, 72.45; H, 6.08; S, 10.75. Found: C, 72.53; H, 5.86; S, 10.82.

1-[2-(4-Chloro-phenylsulfanyl)-phenyl]-pentane-1,4-dione (2c):

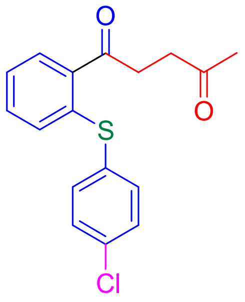

Pale brown oily. **IR** (cm^{-1}) ν3078, 2960, 2912, 2850, 1714, 1668, 1558, 1471, 1458, 1430, 1353, 1261, 1238, 1213, 1164, 1093, 1041, 1011, 983, 959, 824, 755, 745, 678. **^{1}H NMR** (500 MHz, $CDCl_3$) δ2.29 (s, 3H), 2.92 2.95 (t, 2H, J=6.3 Hz), 3.29 3.31 (t, 2H, J=6.2 Hz), 6.89 6.91 (d, 1H, J=9.3 Hz), 7.21 7.24 (m, 1H), 7.29 7.31 (m, 1H), 7.38 7.40 (m, 2H), 7.44 7.47 (m, 2H), 7.93 7.94 (d, 1H, J=9.0 Hz). **^{13}C NMR** (150 MHz, $CDCl_3$) δ 30.1, 33.8, 37.1, 110.0 141.0 (12C), 199.6, 207.2. **MS** (m/z) = 43, 69, 77, 108, 137, 163, 184, 247, 257, 275, 318 (M^+). Anal. Calcd. for $C_{17}H_{15}ClO_2S$: C, 64.04; H, 4.74; Cl, 11.12; S, 10.06. Found: C, 64.12; H, 4.85¸ Cl, 11.02; S, 9.95.

1-[2-(4-Bromo-phenylsulfanyl)-phenyl]-pentane-1,4-dione (2d):

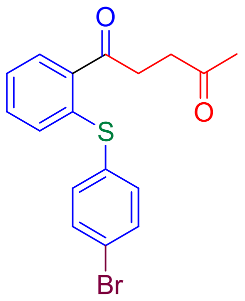

Pale brown oily. **IR** (cm^{-1}) ν 3057, 3027, 2914, 2853, 1709, 1661, 1581, 1558, 1461, 1430, 1359, 1269, 1208, 1162, 1062, 1006, 957, 811, 745, 696. **^{1}H NMR** (500 MHz, $CDCl_3$) δ 2.29 (s, 3H), 2.92 2.95 (t, 2H, J=5.8 Hz), 3.28 3.31 (t, 2H, J=6.5 Hz), 6.91 6.93 (d, 1H, J=9.4 Hz), 7.21 7.24 (m, 1H), 7.29 7.32 (m, 1H), 7.37 7.39 (m, 2H), 7.53 7.56 (m, 2H), 7.92 7.94 (d, 1H, J=9.4 Hz). **^{13}C NMR** (150 MHz, $CDCl_3$) δ 30.1, 33.8, 37.1, 123.3 140.7 (12C), 199.6, 207.2. **MS** (m/z) = 43, 77, 105, 137, 165, 184, 212, 363 (M^+). Anal. Calcd. for $C_{17}H_{15}BrO_2S$: C, 56.21; H, 4.16; Br, 22.00; S, 8.83. Found: C, 56.33; H, 4.15; S, 8.79.

1-[2-(4-Methoxy-phenylsulfanyl)-phenyl]-pentane-1,4-dione (2e):

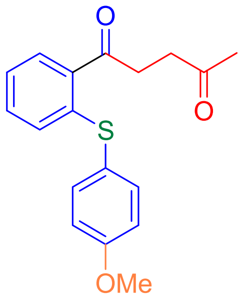

Pale brown oily. **IR** (cm^{-1}) ν 3068, 2996, 2912, 2835, 1712, 1671, 1597, 1499, 1471, 1443, 1356, 1274, 1218, 1195, 1154, 1105, 1031, 993, 883, 844, 762. **^{1}H NMR** (500 MHz, $CDCl_3$) δ 2.30 (s, 3H), 2.93 2.96 (t, 2H, J=5.8 Hz), 3.31 3.34 (t, 2H, J=6.7 Hz), 3.87 (s, 3H), 6.83 6.84 (d, 1H, J=9.4 Hz), 6.96 6.99 (m, 2H), 7.14 7.18 (m, 1H), 7.23 7.27 (m, 1H), 7.46 7.49 (m, 2H), 7.94 7.95 (d, 1H, J=9.5 Hz). **^{13}C NMR** (150 MHz, $CDCl_3$) δ 30.1, 33.6, 37.1, 55.3, 110.0 143.7 (11C), 160.5, 199.3, 207.36. **MS** (m/z) = 43, 65, 77, 109, 137, 163, 171, 200, 243, 253, 314 (M^+). Anal. Calcd. for $C_{18}H_{18}O_3S$: C, 68.76; H, 5.77; S, 10.20. Found: C, 68.57; H, 5.68; S, 10.29.

1-(2-Phenoxy-phenyl)-pentane-1,4-dione (2f):

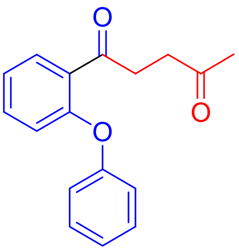

Orange oily. **IR** (cm^{-1}) ν 3060, 2917, 1712, 1671, 1594, 1571, 1471, 1446, 1356, 1269, 1228, 1159, 1108, 1070, 995, 878, 798, 747, 688. **^{1}H NMR** (500 MHz, $CDCl_3$) δ 2.23 (s, 3H), 2.82 2.85 (t, 2H, J=6.3 Hz), 3.32 3.34 (t, 2H, J=6.0 Hz), 6.84 6.95 (m, 2H), 7.05 7.06 (m, 2H), 7.16 7.25 (m, 2H), 7.37 7.45 (m, 2H), 7.84 7.86 (d, 1H, J=9.8 Hz). **^{13}C NMR** (150 MHz, $CDCl_3$) δ 30.0, 37.5, 37.5, 115.29 133.5 (10C), 156.2 156.4 (2C), 199.8, 207.5. **MS** (m/z) = 51, 77, 105, 137, 152, 175, 181, 197, 213, 268 (M^+). Anal. Calcd. for $C_{17}H_{16}O_3$: C, 76.10; H, 6.01. Found: C, 76.16; H, 6.04.

1-(2-p-Tolyloxy-phenyl)-pentane-1,4-dione (2g):

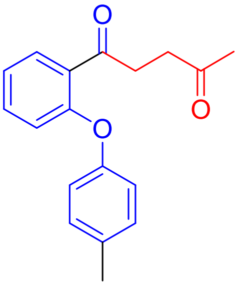

Pale brown oily. **IR** (cm^{-1}) ν3065, 3027, 2922, 1712, 1673, 1594, 1504, 1471, 1448, 1361, 1274, 1226, 1162, 1103, 990, 883, 837, 762. **^{1}H NMR** (500 MHz, $CDCl_3$) δ 2.23 (s, 3H), 2.36 (s, 3H), 2.82 2.84 (t, 2H, J=5.5 Hz), 3.33 3.35 (t, 2H, J=6.2 Hz), 6.86 6.88 (d, 1H, J=8.3 Hz), 6.94 6.97 (m, 2H), 7.12 7.14 (m, 1H), 7.18 7.19 (m, 2H), 7.82 7.84 (d, 1H, J=9.5 Hz). **^{13}C NMR** (150 MHz, $CDCl_3$) δ 20.7, 30.0, 37.5, 37.5, 110.0 133.6 (10C), 153.8, 156.9, 199.8, 207.4. **MS** (m/z) = 51, 77, 109, 121, 152, 165, 174, 197, 211, 227, 244, 282 (M^+). Anal. Calcd. for $C_{18}H_{18}O_3$: C, 76.57; H, 6.43. Found: C, 76.61; H, 6.40.

1-(2-o-Tolyloxy-phenyl)-pentane-1,4-dione (2h):

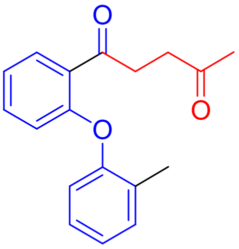

Pale brown oily. **IR** (cm^{-1}) ν 3068, 3027, 2917, 1714, 1673, 1597, 1574, 1471, 1448, 1356, 1228, 1182, 1151, 1110, 1041, 1034, 995, 875, 750. **^{1}H NMR** (500 MHz, $CDCl_3$) δ 2.23 (s, 3H), 2.28 (s, 3H), 2.85 2.87 (t, 2H, J=6.0 Hz), 3.39 3.41 (t, 2H, J=6.8 Hz), 6.66 6.70 (d, 1H, J=19.3 Hz), 6.92 6.94 (d, 1H, J=8.1 Hz), 7.06 7.15 (m, 3H), 7.29 7.39 (m, 2H), 7.84 7.85 (d, 1H, J=7.8 Hz). **^{13}C NMR** (150 MHz, $CDCl_3$) δ 16.2, 30.0, 37.5, 37.7, 116.8 133.5 (10C), 153.5, 157.01, 199.8, 207.5**.** **MS** (m/z) = 65, 77, 91, 128, 152, 184, 197, 218, 247, 281 (M^+). Anal. Calcd. for $C_{18}H_{18}$ O_3: C, 76.57; H, 6.43. Found: C, 76.51; H, 6.47.

1-(2-m-Tolyloxy-phenyl)-pentane-1,4-dione (1i):

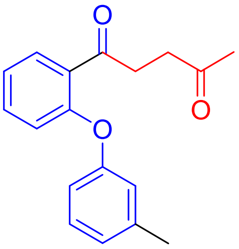

Pale brown oily. **IR** (cm^{-1}) ν 3061, 3024, 2914, 1714, 1676, 1594, 1574, 1471, 1448, 1359, 1246, 1215, 1156, 1139, 1105, 993, 936, 765, 688. **^{1}H NMR** (500 MHz, $CDCl_3$) δ 2.23 (s, 3H), 2.36 (s, 3H), 2.82 2.84 (t, 2H,

J=7.1 Hz), 3.32 3.34 (t, 2H, J=7.1 Hz), 6.84 6.91 (m, 3H), 6.98 6.99 (d, 1H, J=7.6 Hz), 6.15 6.26 (m, 2H), 7.40 7.44 (m, 1H), 7.83 7.85 (d, 1H, J=9.5 Hz). **^{13}C NMR** (150 MHz, $CDCl_3$) δ 21.3, 30.0, 37.5, 37.5, 116.1 133.4 (9C), 140.3, 156.1, 156.5, 199.7, 207.4. **MS** (m/z) = 43, 65, 77, 91, 105, 121, 175, 211, 239, 264, 282 (M^+). Anal. Calcd. for $C_{18}H_{18}O_3$: C, 76.57; H, 6.43. Found: C, 76.62; H, 6.48.

1-[2-(4-Chloro-phenoxy)-phenyl]-pentane-1,4-dione (2j):

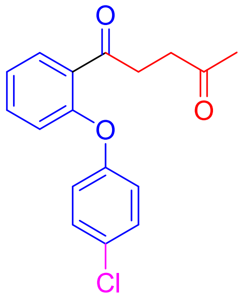

Pale brown oily. **IR** (cm^{-1}) ν 3060, 2914, 1712, 1673, 1597, 1571, 1474, 1448, 1356, 1233, 1162, 1087, 1006, 829, 760. **^{1}H NMR** (500 MHz, $CDCl_3$) δ 2.23 (s, 3H), 2.82 2.85 (t, 2H, J=6.0 Hz), 3.26 3.29 (t, 2H, J=5.4 Hz), 6.89 6.91 (m, 1H), 6.97 7.00 (m, 2H), 7.19 7.22 (m, 1H), 7.32 7.35 (m, 2H), 7.43 7.47 (m, 1H), 7.83 7.85 (d, 1H, J=9.6 Hz). **^{13}C NMR** (150 MHz, $CDCl_3$) δ 30.0, 34.2, 37.3, 119.2 133.5 (10C), 155.0, 155.8, 199.5, 207.3. **MS** (m/z) = 43, 69, 77, 108, 137, 163, 184, 247, 257, 275, 318 (M^+). Anal. Calcd. for $C_{17}H_{15}ClO_3$: C, 67.44; H, 4.99; Cl, 11.71. Found: C, 67.51; H, 5.14; Cl, 11.63.

1-[2-(4-Fluoro-phenoxy)-phenyl]-pentane-1,4-dione (2k):

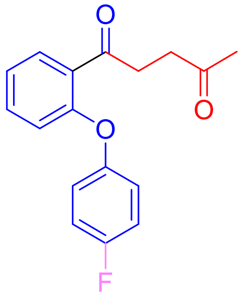

Pale brown oily. **IR** (cm^{-1}) ν3070, 2912, 1712, 1673, 1597, 1502, 1474, 1443, 1353, 1272, 1208, 1182, 1156, 1108, 1090, 995, 847, 816, 765. **^{1}H NMR** (500 MHz, $CDCl_3$) δ 2.23 (s, 3H), 2.83 2.86 (t, 2H, J=6.1 Hz), 3.30 3.33 (t, 2H, J=6.4 Hz), 6.84 6.85 (d, 1H, J=7.1 Hz), 7.01 7.10 (m, 4H), 7.15 7.18 (m, 1H), 7.40 7.44 (m, 1H), 7.82 7.84 (d, 1H, J=9.5 Hz). **^{13}C NMR** (150 MHz, $CDCl_3$) δ 30.0, 37.4, 37.4, 116.5 133.5 (9C), 156.6, 158.2, 160.1, 199.64, 207.37. **^{19}F NMR** (470 MHz, $CDCl_3$) δ -121.07 (s). MS (m/z) = 43, 69, 77, 107, 133, 175, 186, 215, 243, 268, 286 (M^+). Anal. Calcd. for $C_{17}H_{15}FO_3$: C, 71.32; H, 5.28; F, 6.64. Found: C, 71.43; H, 5.16; F, 6.58.

1-[2-(4-Bromo-phenoxy)-phenyl]-pentane-1,4-dione (2l):

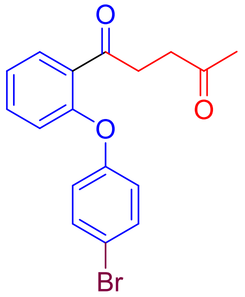

Pale brown oily. **IR** (cm^{-1}) ν 3057, 3027, 2914, 2853, 1709, 1661, 1581, 1558, 1461, 1430, 1359, 1269, 1208, 1162, 1062, 1006, 957, 811, 745, 696. **^{1}H NMR** (500 MHz, $CDCl_3$) δ 2.29 (s, 3H), 2.92 2.95 (t, 2H, J=5.8 Hz), 3.28 3.31 (t, 2H, J=6.5 Hz), 6.91 6.93 (d, 1H, J=9.4 Hz), 7.21 7.24 (m, 1H), 7.29 7.32 (m, 1H), 7.37 7.39 (m, 2H), 7.53

7.56 (m, 2H), 7.92 7.94 (d, 1H, J=9.4 Hz). **^{13}C NMR** (150 MHz, $CDCl_3$) δ 30.1, 33.8, 37.1, 123.3 140.7 (12C), 199.6, 207.2. **MS** (m/z) = 43, 76, 108, 137, 184, 211, 240, 293, 301, 363 (M^+), 365. Anal. Calcd. for $C_{17}H_{15}BrO_2S$: C, 58.81; H, 4.35; Br, 23.01. Found: C, 58.88; H, 4.30; Br, 22.85.

1-[2-(Naphthalen-1-yloxy)-phenyl]-pentane-1,4-dione (2m):

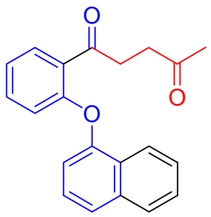

Brown oily. **IR** (cm^{-1}) ν 3052, 2953, 2914, 1712, 1676, 1599, 1504, 1474, 1464, 1446, 1353, 1246, 1220, 1156, 962, 911, 811, 752. **^{1}H NMR** (500 MHz, $CDCl_3$) δ2.21 (s, 3H), 2.81 2.84 (t, 2H, J=7.7 Hz), 3.35 3.37 (t, 2H, J=6.3 Hz), 6.97 6.98 (d, 1H, J=9.5 Hz), 7.20 7.23 (m, 1H), 7.28 7.36 (m, 3H), 7.43 7.51 (m, 3H), 7.73 7.74 (d, 1H, J=8.1 Hz), 7.86 7.90 (m, 2H). **^{13}C NMR** (150 MHz, $CDCl_3$) δ 30.00, 37.48, 37.51, 114.55 134.26 (14C), 154.15 156.23 (2C), 199.69, 207.37. **MS** (m/z) = 43, 77, 94, 128, 165, 174, 247, 257, 300, 318 (M^+). Anal. Calcd. for $C_{21}H_{18}O_3$: C, 79.22; H, 5.70. Found: C, 79.11; H, 5.81.

1-[2-(4-Methoxy-phenoxy)-phenyl]-pentane-1,4-dione (2n):

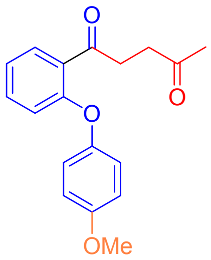

Brown oily. **IR** (cm^{-1}) ν 3068, 2999, 2914, 2832, 1714, 1671, 1504, 1474, 1446, 1356, 1274, 1218, 1192, 1156, 1100, 1029, 993, 875, 844, 760. **^{1}H NMR** (500 MHz, $CDCl_3$) δ 2.24 (s, 3H), 2.83 2.86 (t, 2H, J=6.2 Hz), 3.35 3.38 (t, 2H, J=6.2 Hz), 3.83 (s, 3H), 6.80 6.82 (d, 1H, J=8.3 Hz), 6.91 6.94 (m, 2H), 7.00 7.03 (m, 2H), 7.09 7.12 (m, 1H), 7.37 7.40 (m, 1H), 7.81 7.83 (d, 1H, J=9.8 Hz). **^{13}C NMR** (150 MHz, $CDCl_3$) δ 30.0, 37.5, 37.6, 55.6, 115.0 133.4 (9C), 149.1, 156.3, 157.6, 199.9, 207.5. **MS** (m/z) = 51, 77, 109, 184, 195, 227, 247, 257, 277, 280, 298 (M^+). Anal. Calcd. for $C_{18}H_{18}O_4$: C, 72.47; H, 6.08. Found: C, 72.53; H, 6.12.

2-Methyl-5-(2-p-tolyloxy-phenyl)-furan (4a) :

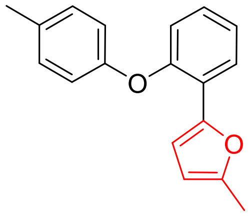

Pale brown oily. **IR** (cm^{-1}) ν 3039, 2948, 2911, 1684, 1576, 1502, 1482, 1436, 1229, 1192, 1019, 837, 793, 748. **^{1}H NMR** (400 MHz, $CDCl_3$) δ 2.34 (s, 3H), δ 2.34 (s, 3H), 6.04 (s, 1H), 6.80 (d, *J* = 3.2 Hz, 1H), 6.90 6.93 (m, 3H), 7.13 7.16 (m, 4H), 7.91 (d, *J* = 9.5 Hz, 1H). **^{13}C NMR** (101 MHz, $CDCl_3$) δ 154.63, 152.45, 151.35, 147.91, 138.56, 132.60, 130.25, 127.32, 125.75, 123.48, 119.24, 118.89, 118.33, 111.32, 108.05, 20.6, 13.68. **MS**

(m/z) = 44, 65, 77, 89, 102, 115, 128, 139, 152, 165, 178, 189, 195, 205, 221, 232, 129, 264 (M^+). Anal. Calcd. for $C_{18}H_{16}O_2$: C, 81.79; H, 6.10. Found: C, 81.73; H, 6.12.

2-Methyl-1-phenyl-5-(2-p-tolyloxy-phenyl)-1H-pyrrole (4b) :

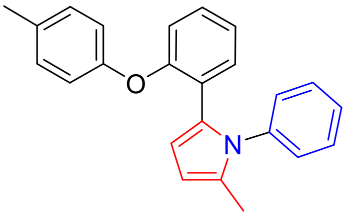

Pale brown oily. **IR** (cm^{-1}) ν 3061, 3024, 2919, 2850, 1598, 1576, 1497, 1443, 1396, 1236, 1204, 1042, 771, 741, 699. **^{1}H NMR** (400 MHz, $CDCl_3$) δ 2.17 (d, J = 7.6 Hz, 3H), 2.32 (d, J = 7.5 Hz, 3H), 6.12 (d, J = 3.5 Hz, 1H), 6.34 (d, J = 5.1 Hz, 1H), 6.45 6.63 (m, 3H), 6.80 7.12 (m, 6H), 7.26 7.35 (m, 4H). **^{13}C NMR** (101 MHz, $CDCl_3$) δ 155.08, 154.21, 139.56, 132.54, 132.14, 130.71, 129.88 128.52 (6C), 128.00, 127.95, 126.68, 124.92, 122.17, 119.30, 117.09, 109.98, 107.46, 20.70, 13.55. **MS** (m/z) = 44, 77, 91, 118, 152, 178, 189, 205, 230, 246, 281, 307, 322, 339 (M^+). Anal. Calcd. for $C_{24}H_{21}NO$: C, 82.92; H, 6.24; N, 4.13. Found: C, 82.83; H, 6.25; N, 4.12.

2-Methyl-1-naphthalen-1-yl-5-(2-p-tolyloxy-phenyl)-1H-pyrrole (4c) :

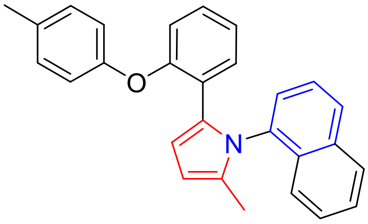

Brown oily. **IR** (cm^{-1}) ν 3052, 2919, 2850, 1596, 1579, 1502, 1468, 1409, 1226, 1165, 1017, 803, 773, 751. **^{1}H NMR** (400 MHz, $CDCl_3$) δ 1.94 (s, 3H), δ 2.30 (s, 3H), 6.20 (s, 1H), 6.43 6.48 (m, 2H), 6.57 (d, J = 8.2 Hz, 1H), 6.74 (d, J = 7.5 Hz, 1H), 6.96 6.99 (m, 3H), 7.09 (d, J = 7.6 Hz, 1H), 7.27 7.42 (m, 6H), 7.83 (dd, J_1 = 3.3 Hz, J_2 = 7.6 Hz, 1H). **^{13}C NMR** (101 MHz, $CDCl_3$) δ 155.06, 154.60, 136.19, 133.99, 132.14, 132.05, 131.77, 130.74 129.76 (6C), 128.08, 127.77, 126.81, 126.73, 126.11, 125.11, 125.01, 124.12, 122.09, 118.85, 117.73, 110.16, 107.06, 20.64, 12.97. **MS** (m/z) = 44, 65, 77, 91, 115, 127, 168, 207, 221, 254, 296, 331, 347, 357, 388, 389 (M^+). Anal. Calcd. for $C_{28}H_{23}NO$: C, 86.34; H, 5.95; N, 3.60. Found: C, 86.37; H, 6.02; N, 3.55.

References:

[1] (a) Yeager, G. W.; Schissel, D. N. An Employed Synthon Approach to the Synthesis of 2-Aryloxyphenols. *Synthesis* **1995**, *1*, 28. (b) Yildiz, T.; Küçük, H. B. An Organocatalytic Method for the Synthesis of Some Novel Xanthene Derivatives by the Intramolecular Friedel-Crafts Reaction. *RSC Adv.* **2017**, *7* (27), 16644–16649. **(c)**Yildiz, T. Synthesis of New Thioxanthenes by Organocatalytic Intramolecular Friedel–Crafts Reaction. *Synth. Commun.* **2018**, *48* (17), 2177–2188.

6. NMR Spectra of all the reported compounds:

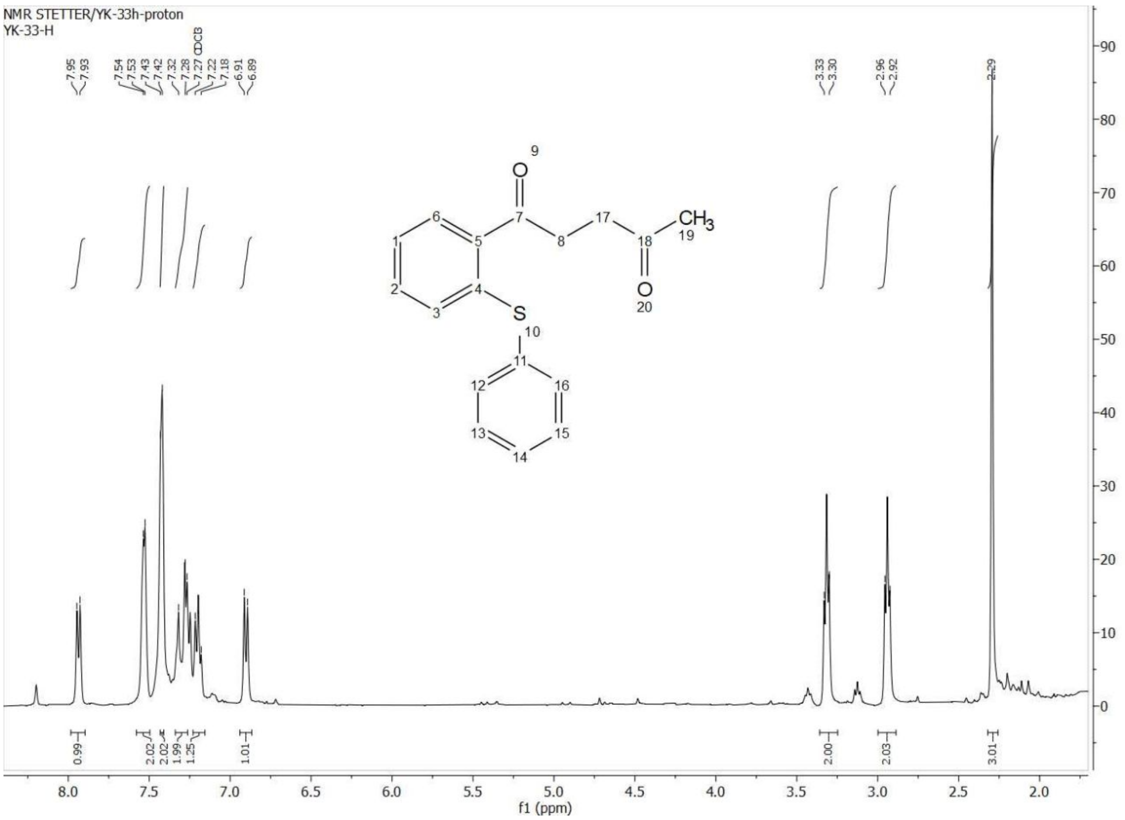

Scheme S6. ^{1}H NMR spectrum of **2a**.

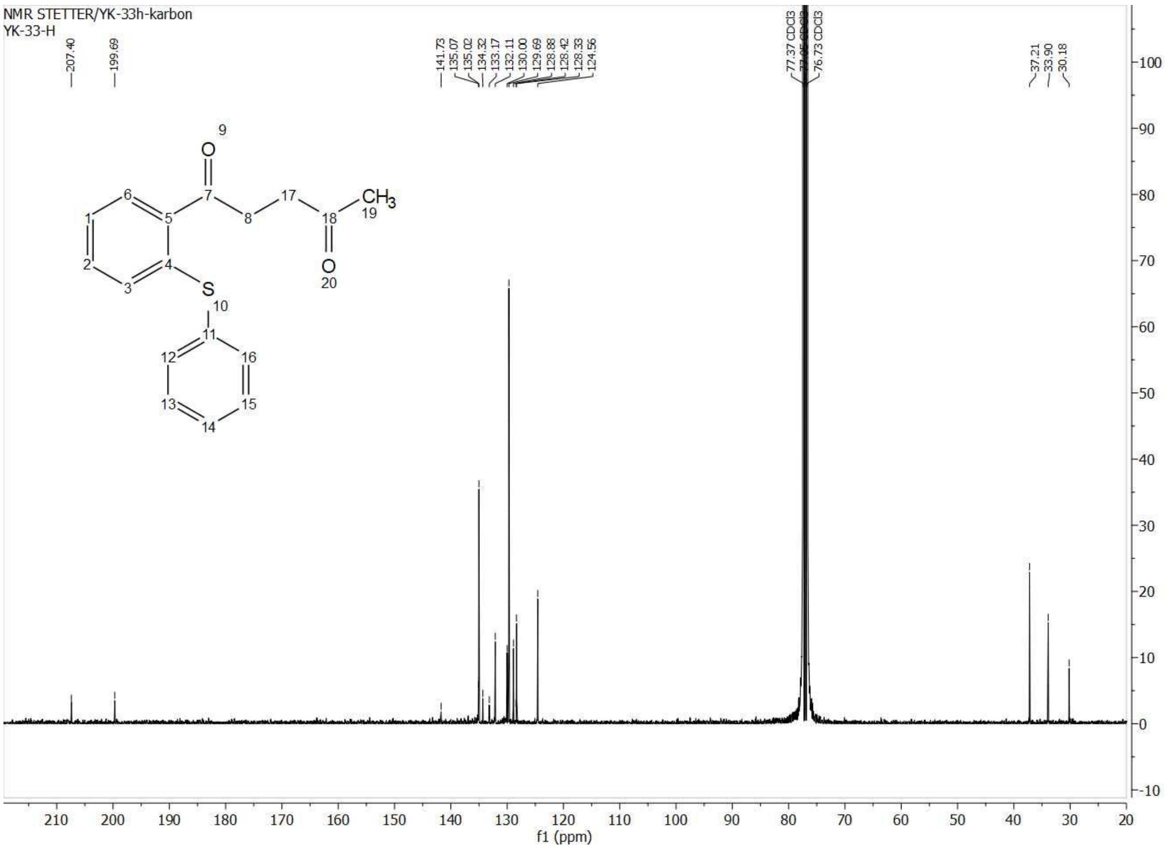

Scheme S7. ^{13}C NMR spectrum of **2a**.

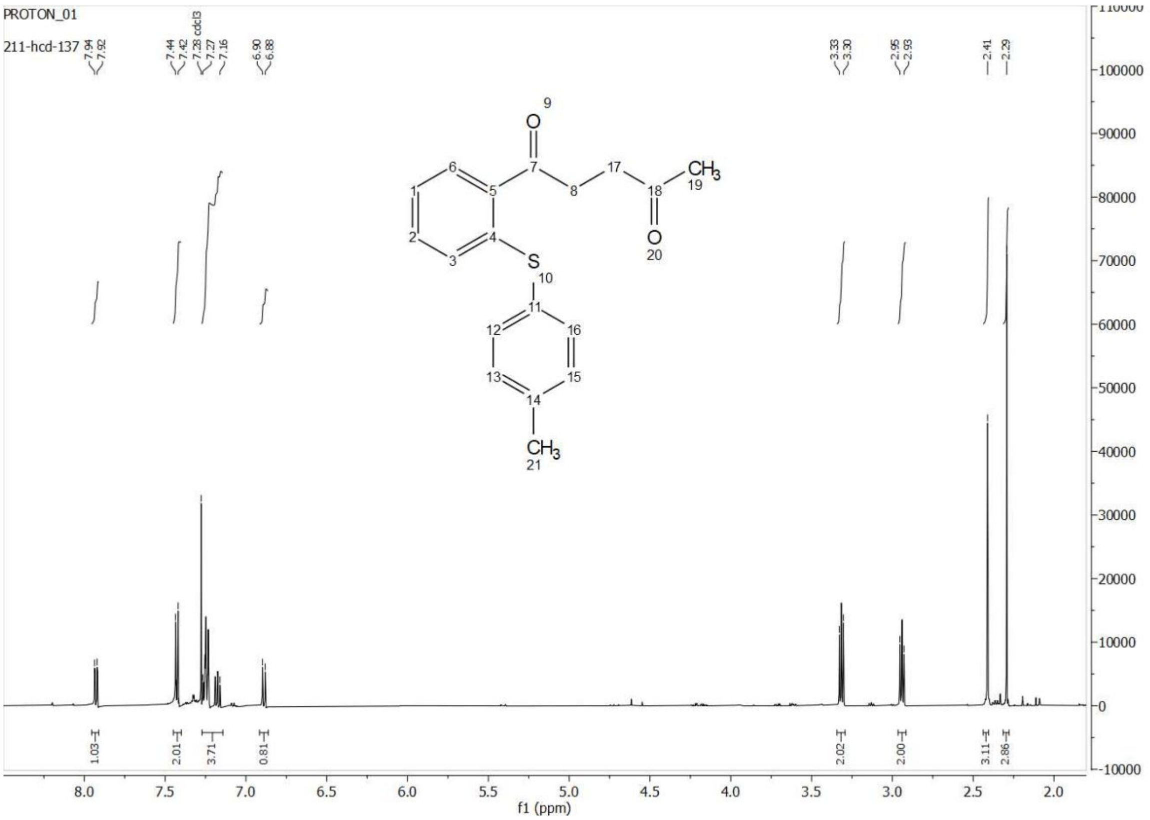

Scheme S8. ^{1}H NMR spectrum of **2b**.

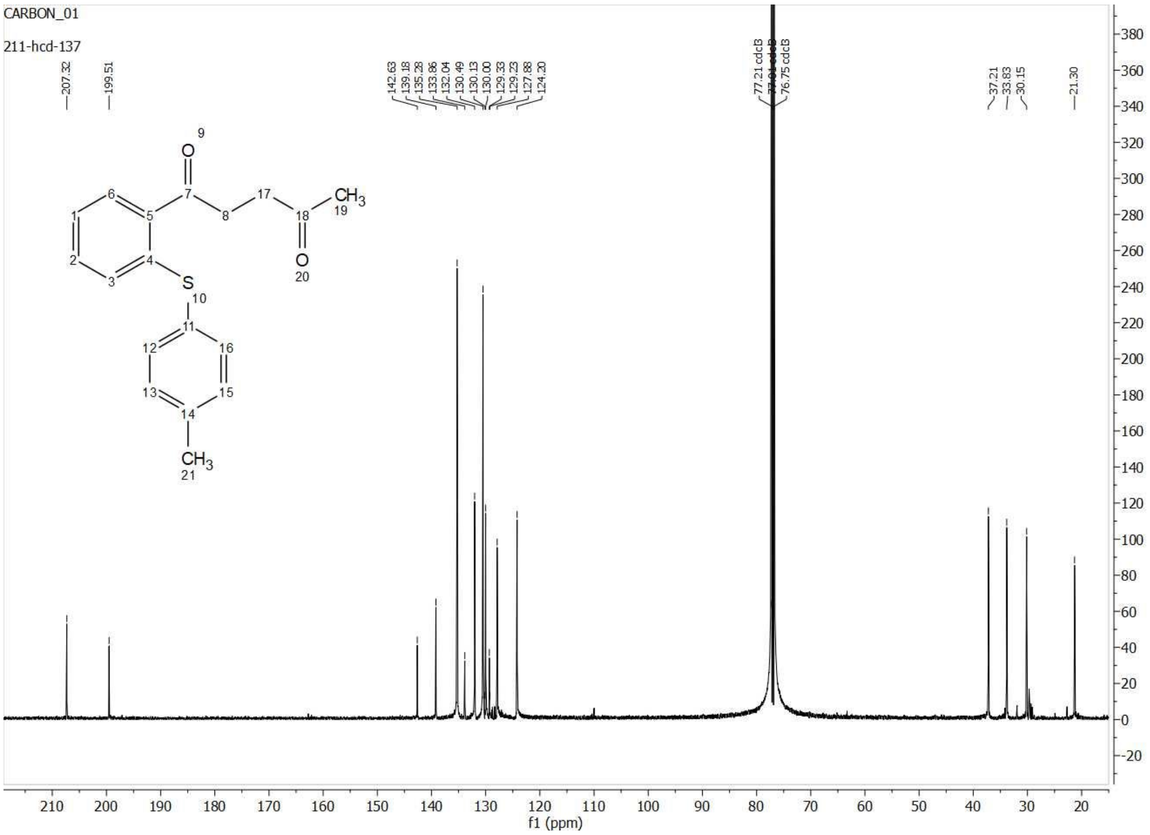

Scheme S9. ^{13}C NMR spectrum of **2b**.

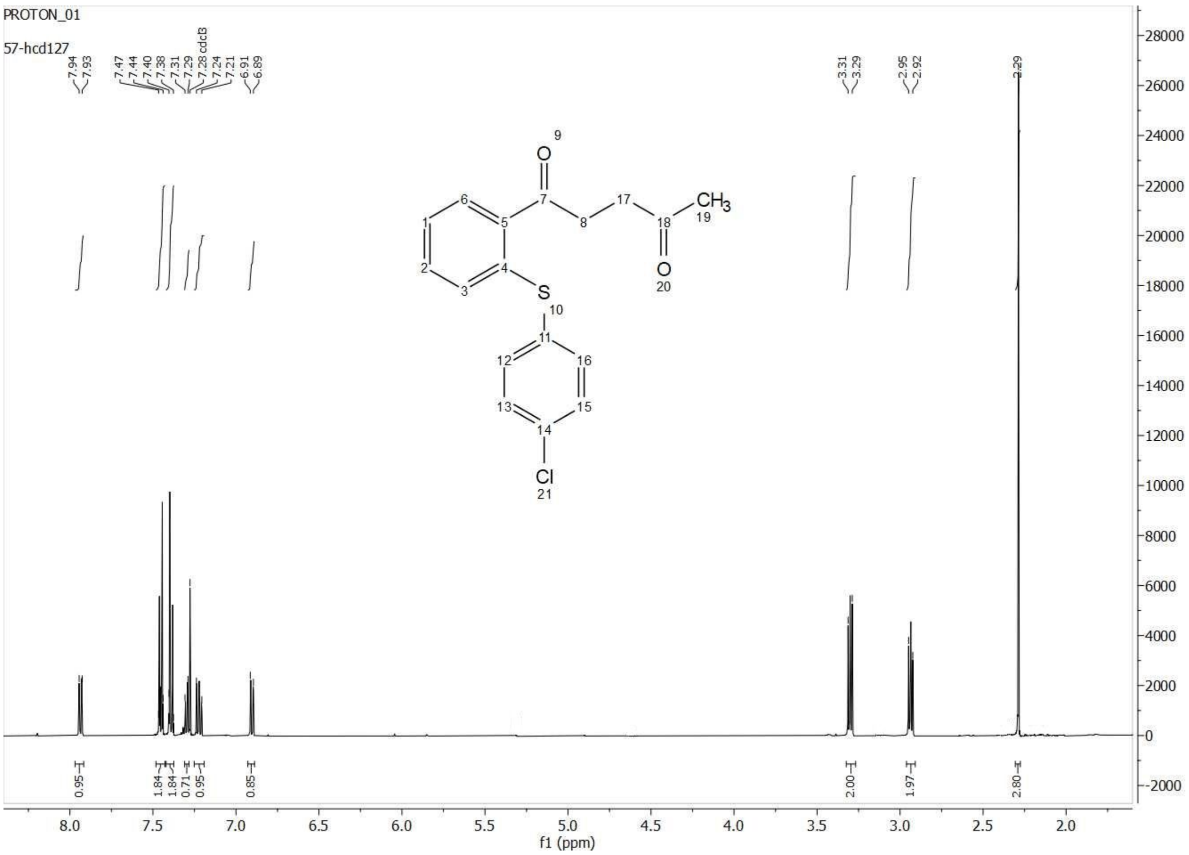

Scheme S10. ^{1}H NMR spectrum of **2c**.

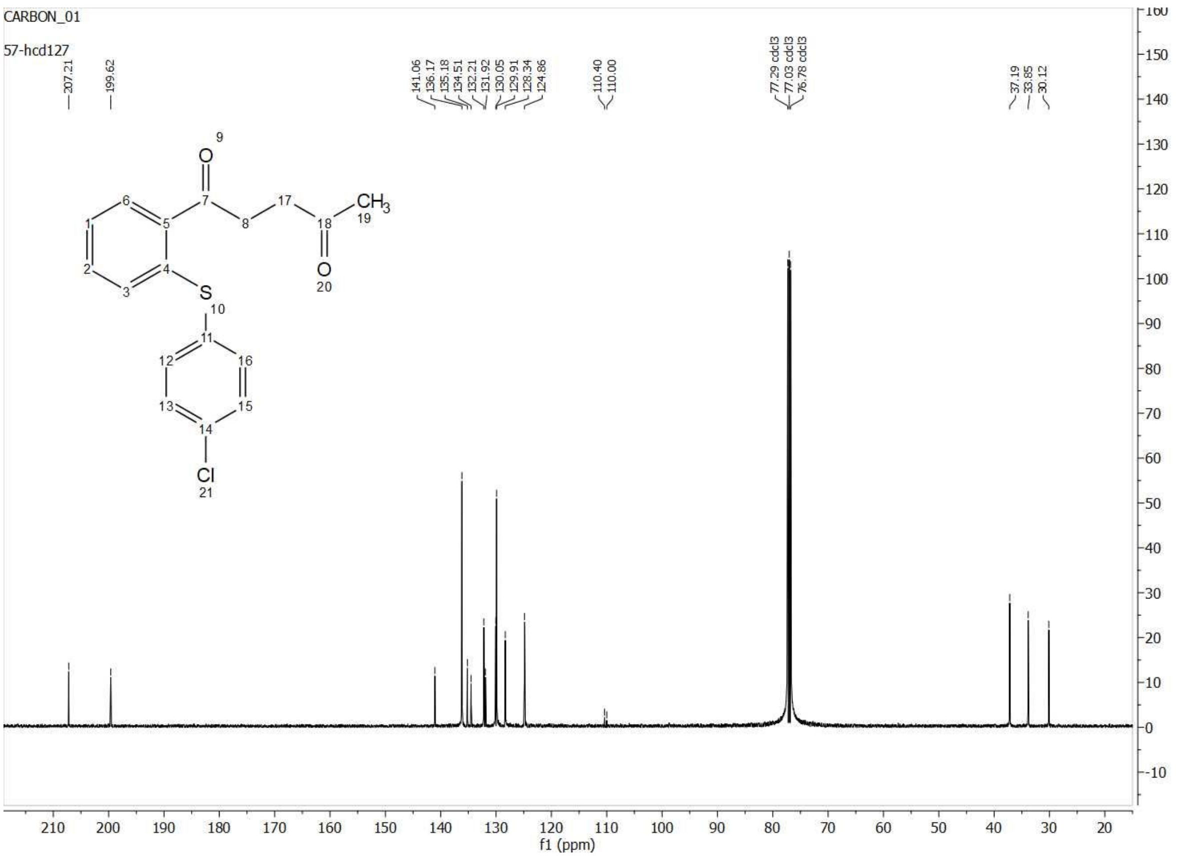

Scheme S11. ^{13}C NMR spectrum of **2c**.

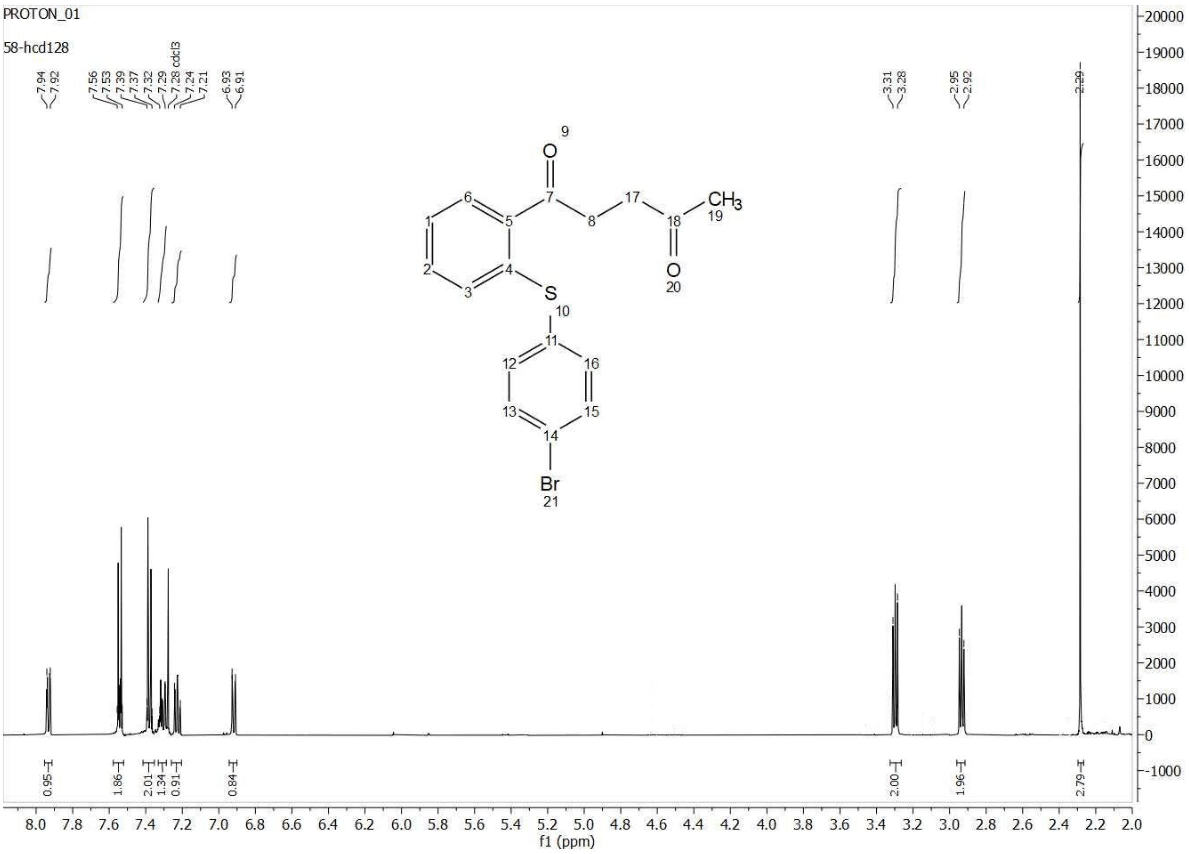

Scheme S12. ^{1}H NMR spectrum of **2d**.

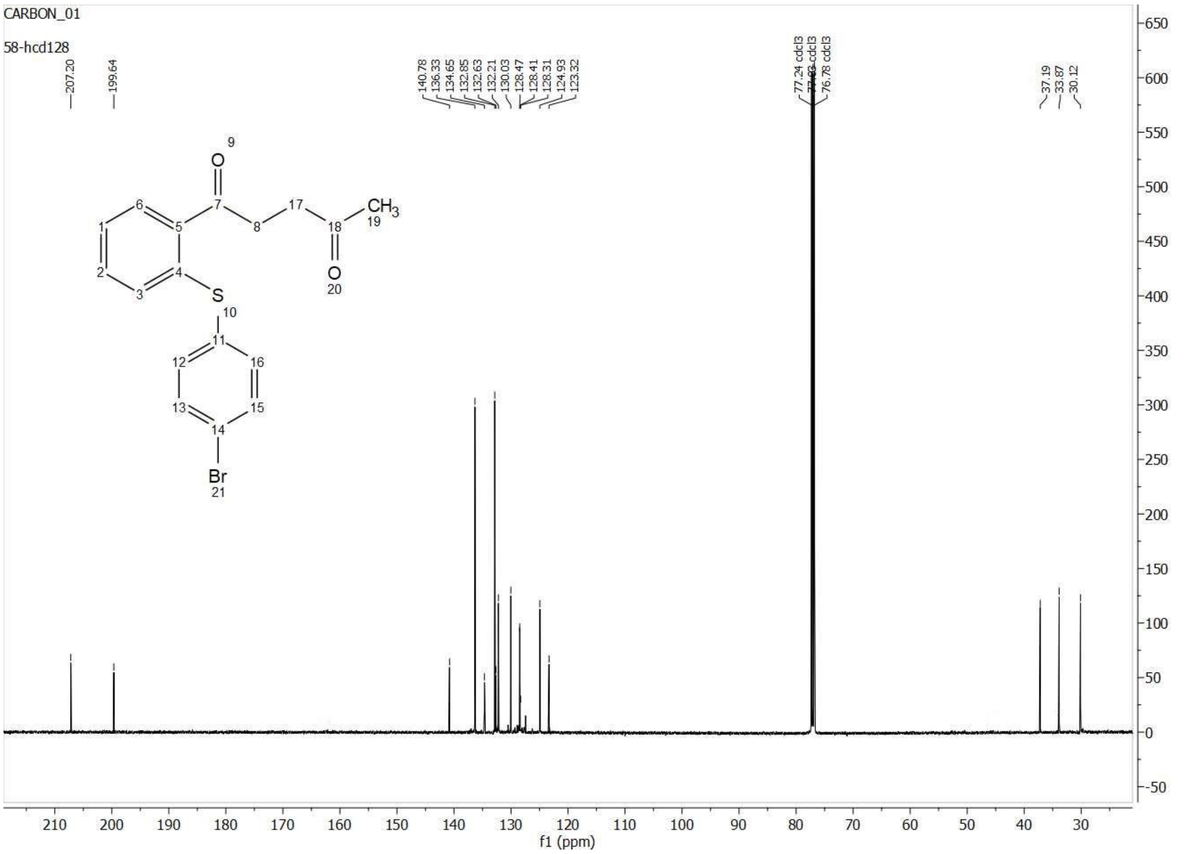

Scheme S13. ^{13}C NMR spectrum of **2d**.

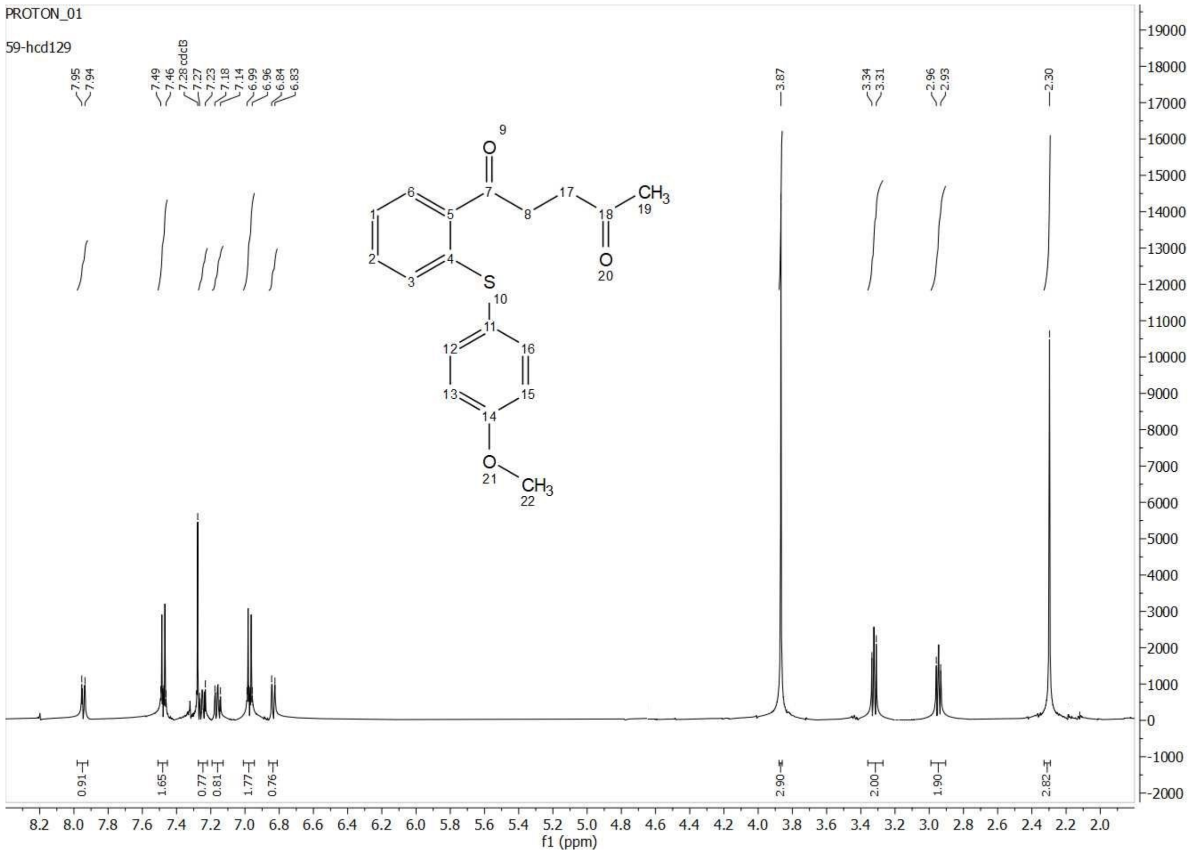

Scheme S14. ^{1}H NMR spectrum of **2e**.

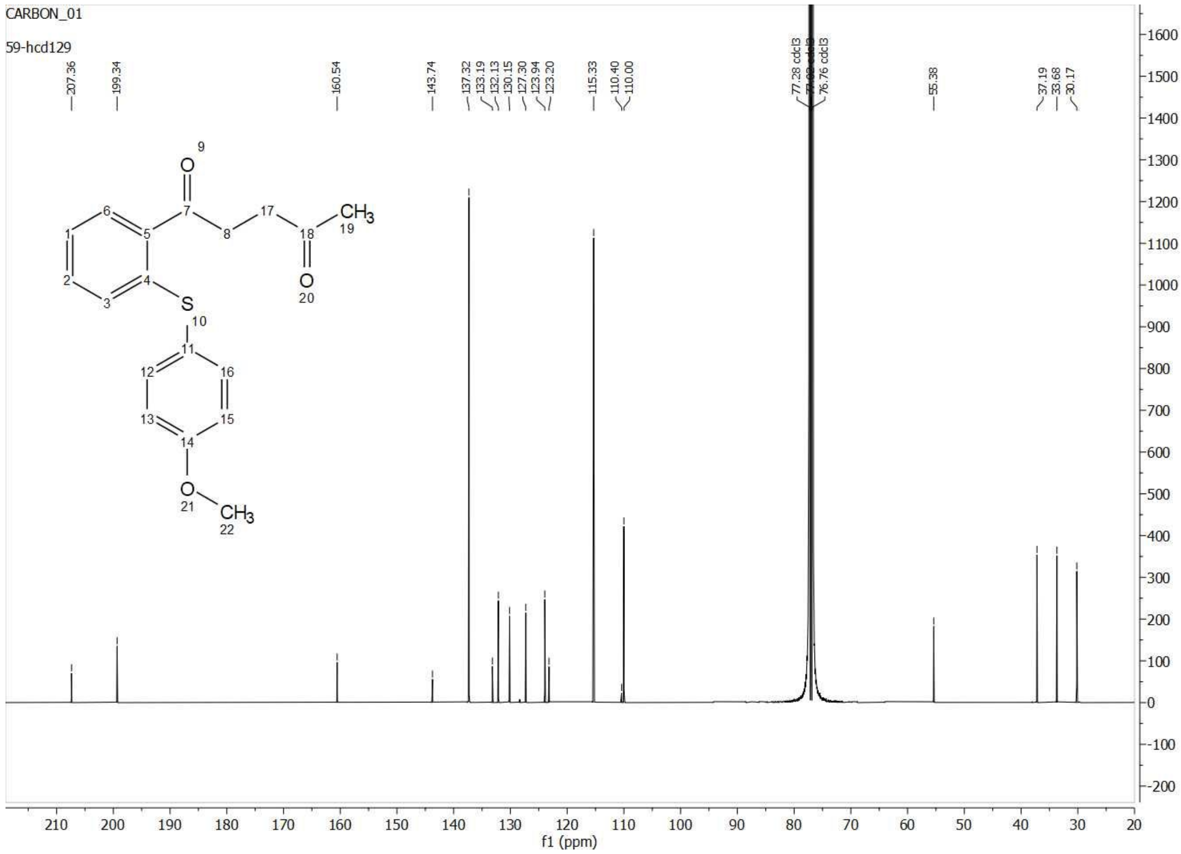

Scheme S15. ^{13}C NMR spectrum of **2e**.

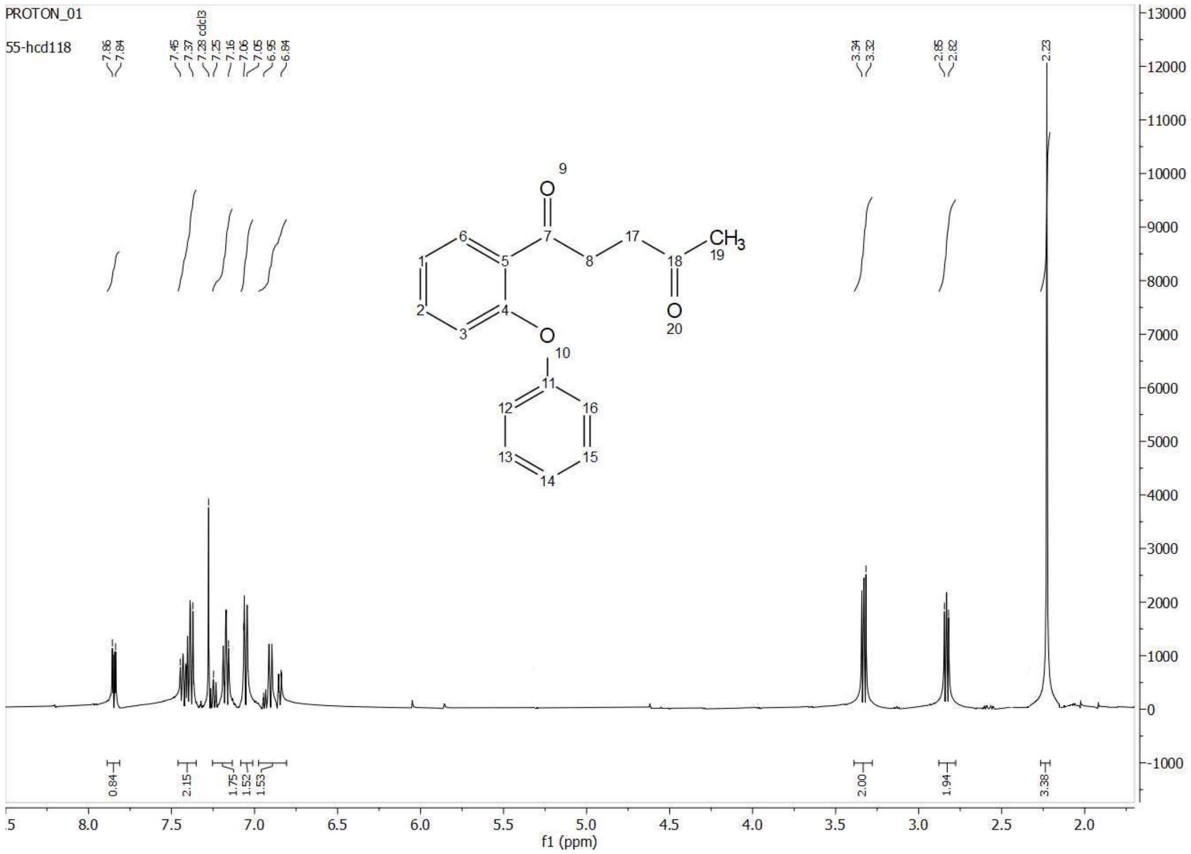

Scheme S16. ^{1}H NMR spectrum of **2f**.

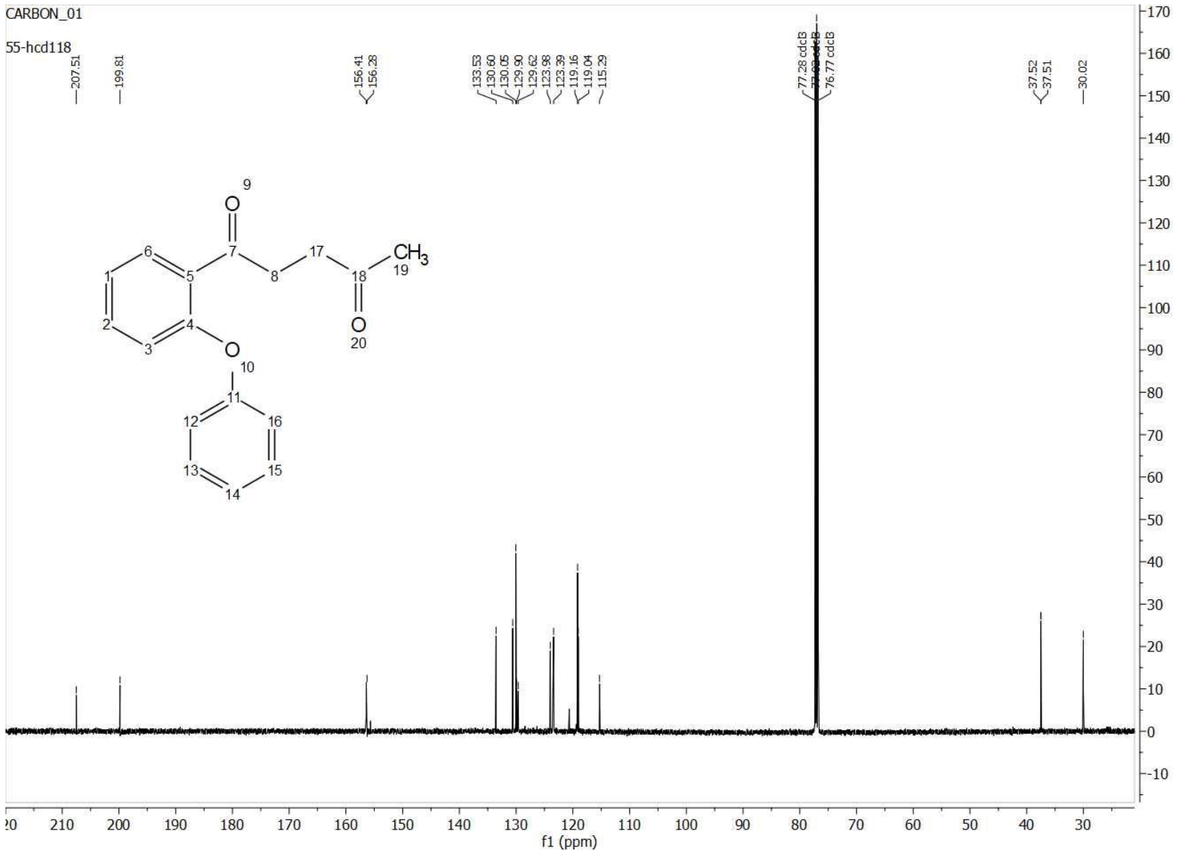

Scheme S17. ^{13}C NMR spectrum of **2f**.

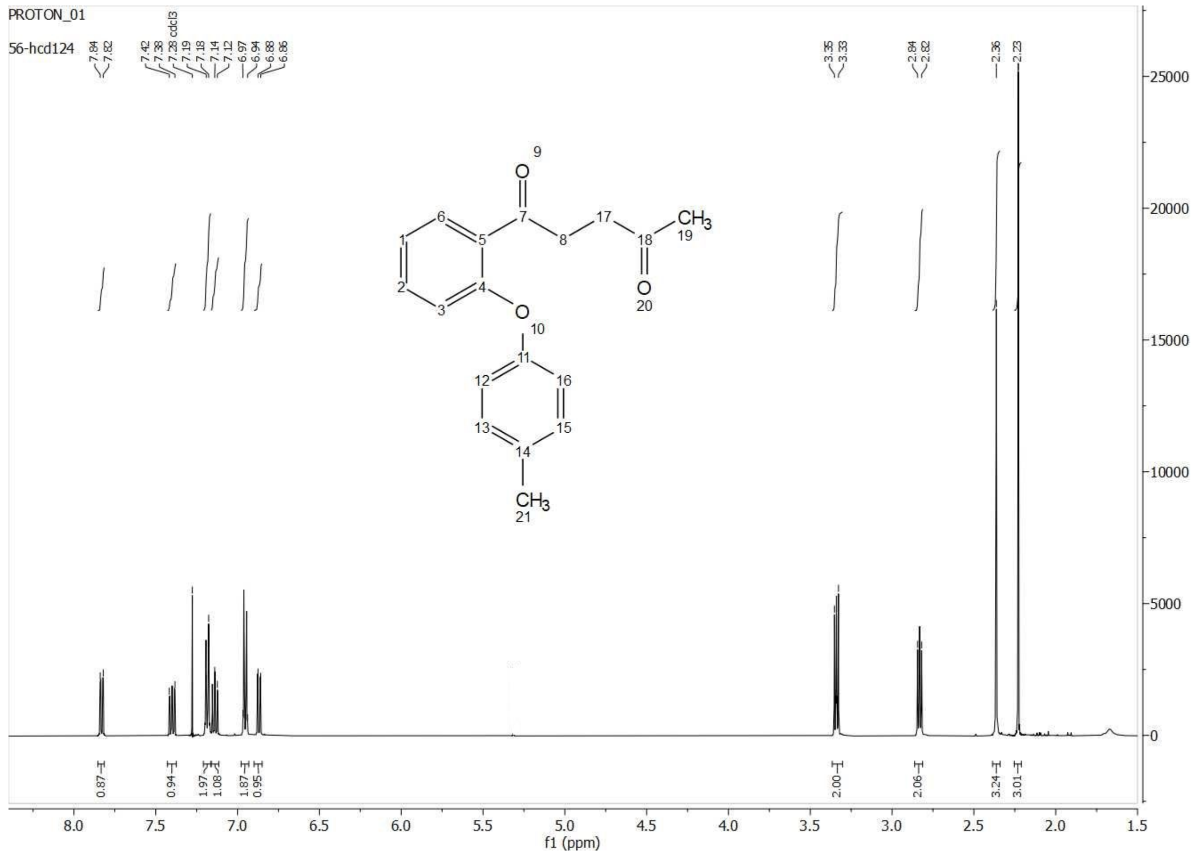

Scheme S18. ^{1}H NMR spectrum of **2g**.

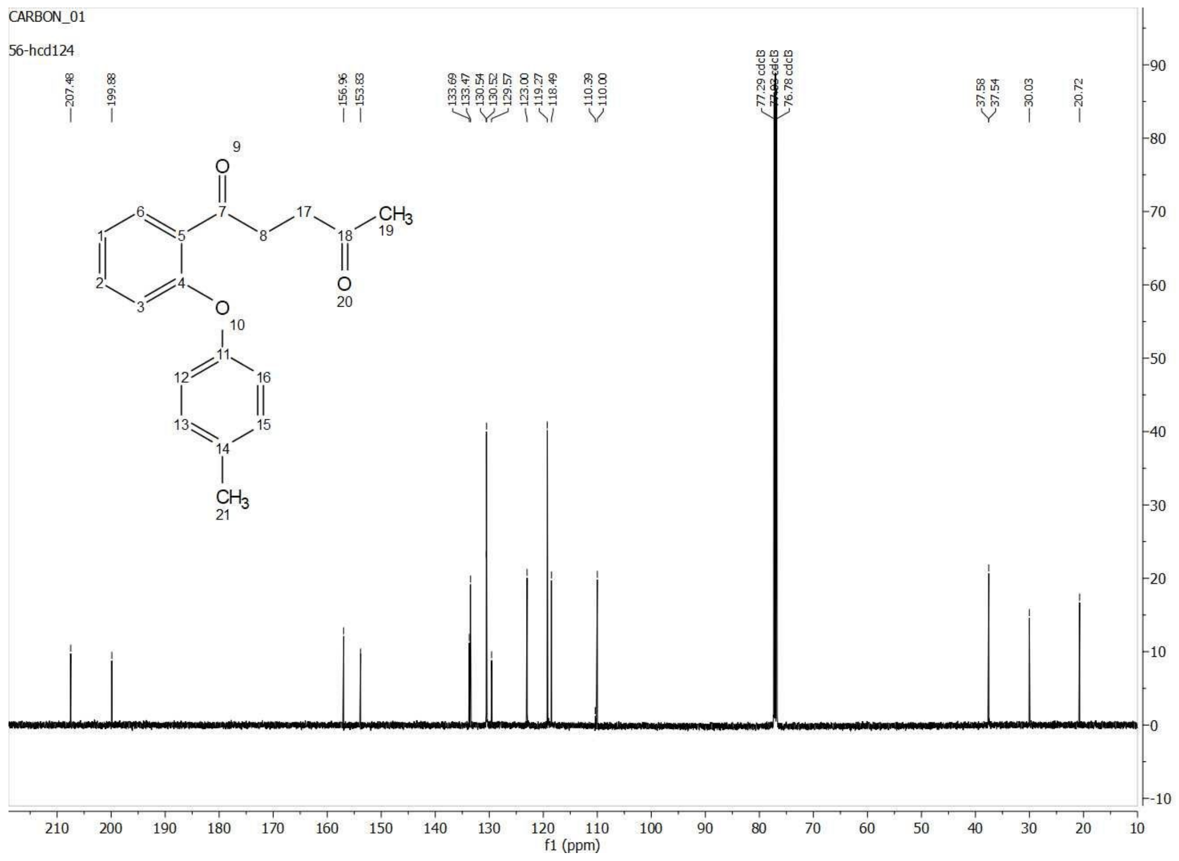

Scheme S19. ^{13}C NMR spectrum of **2g**.

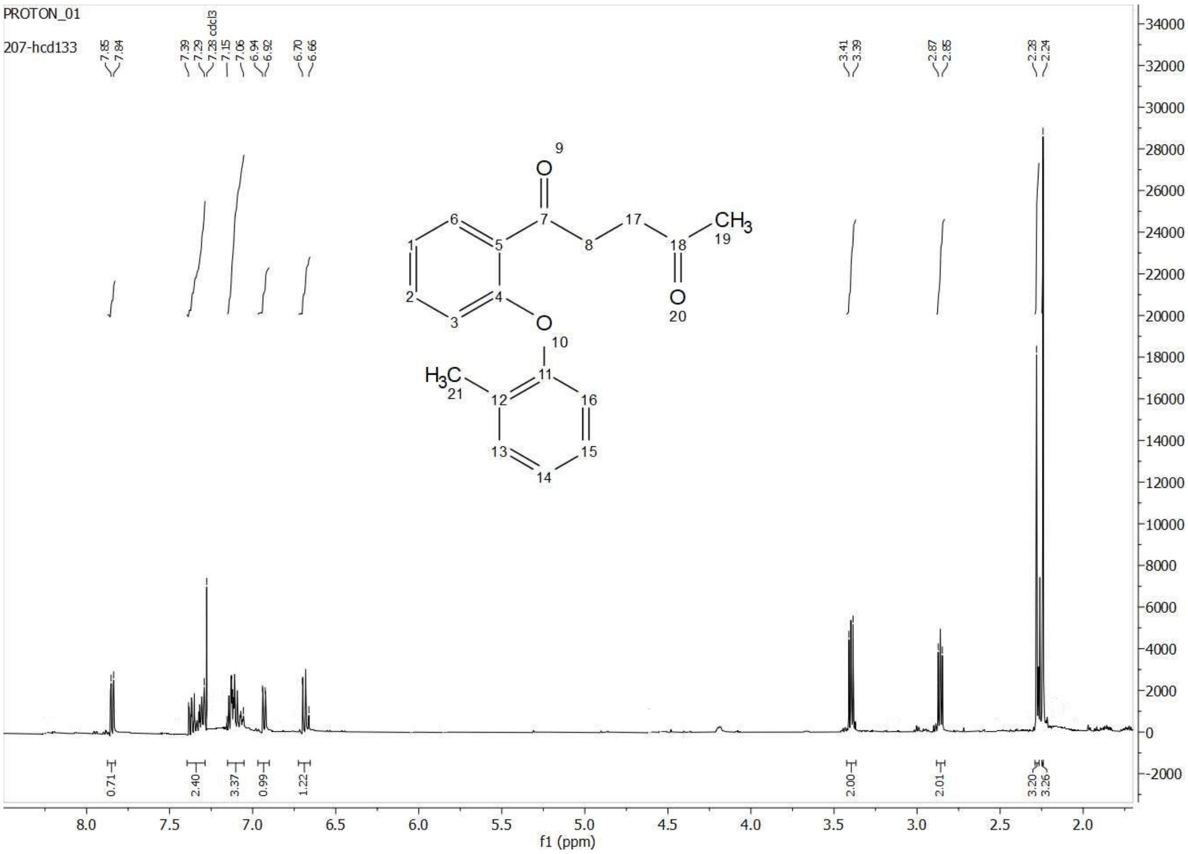

Scheme S20. ^{1}H NMR spectrum of **2h**.

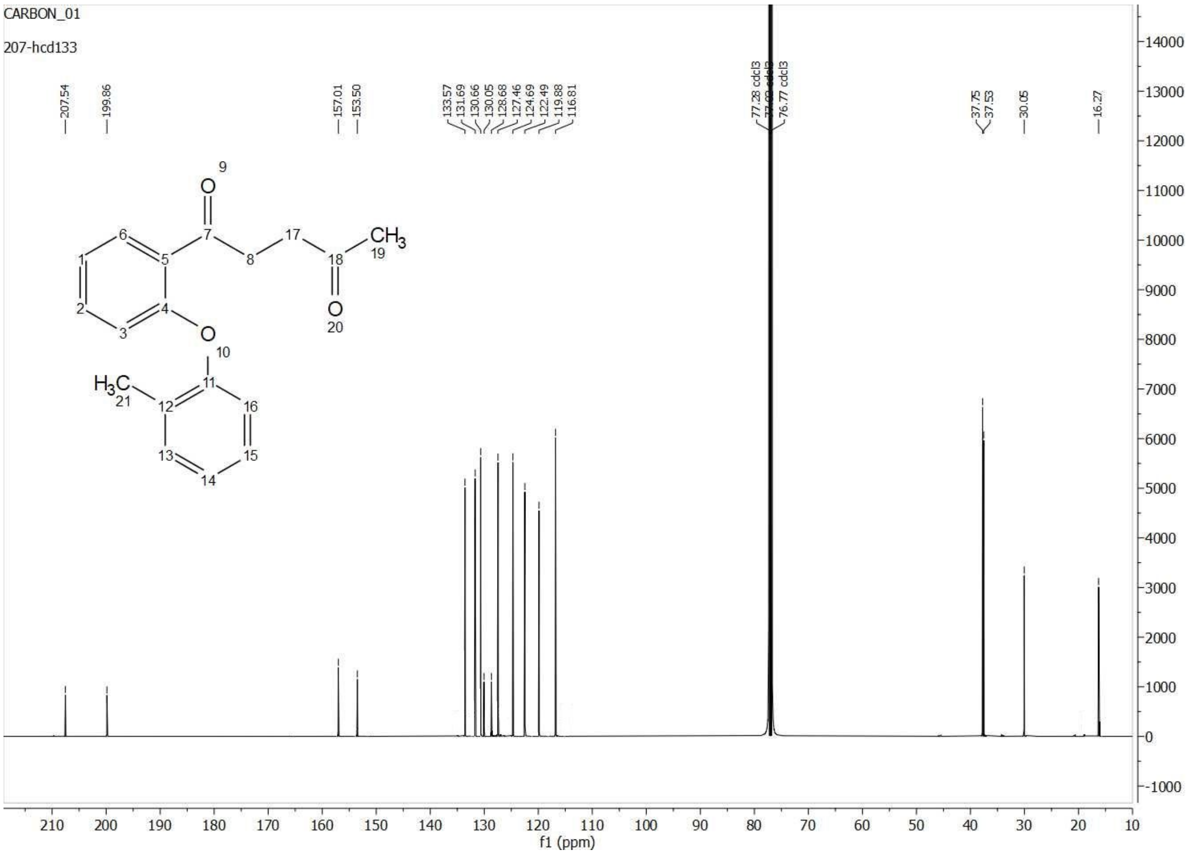

Scheme S21. ^{13}C NMR spectrum of **2h**.

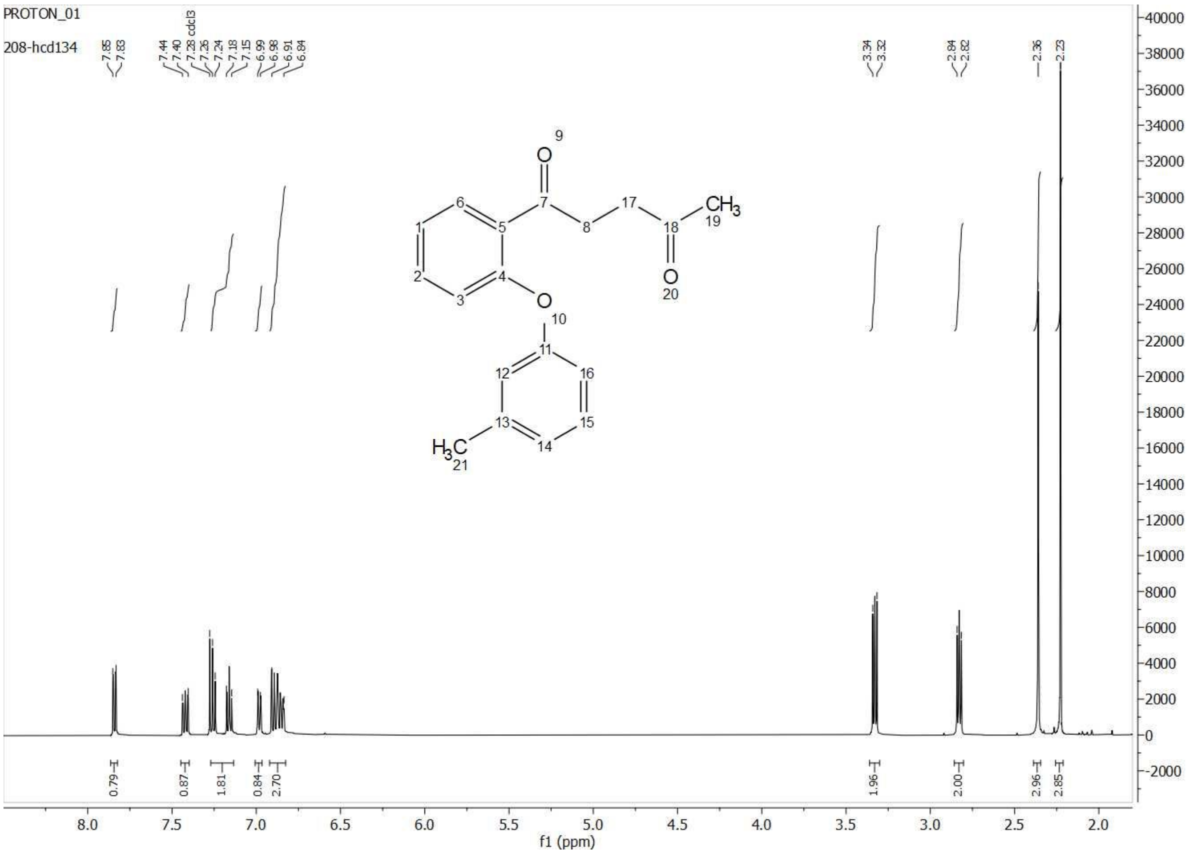

Scheme S22. ^{1}H NMR spectrum of **2i**.

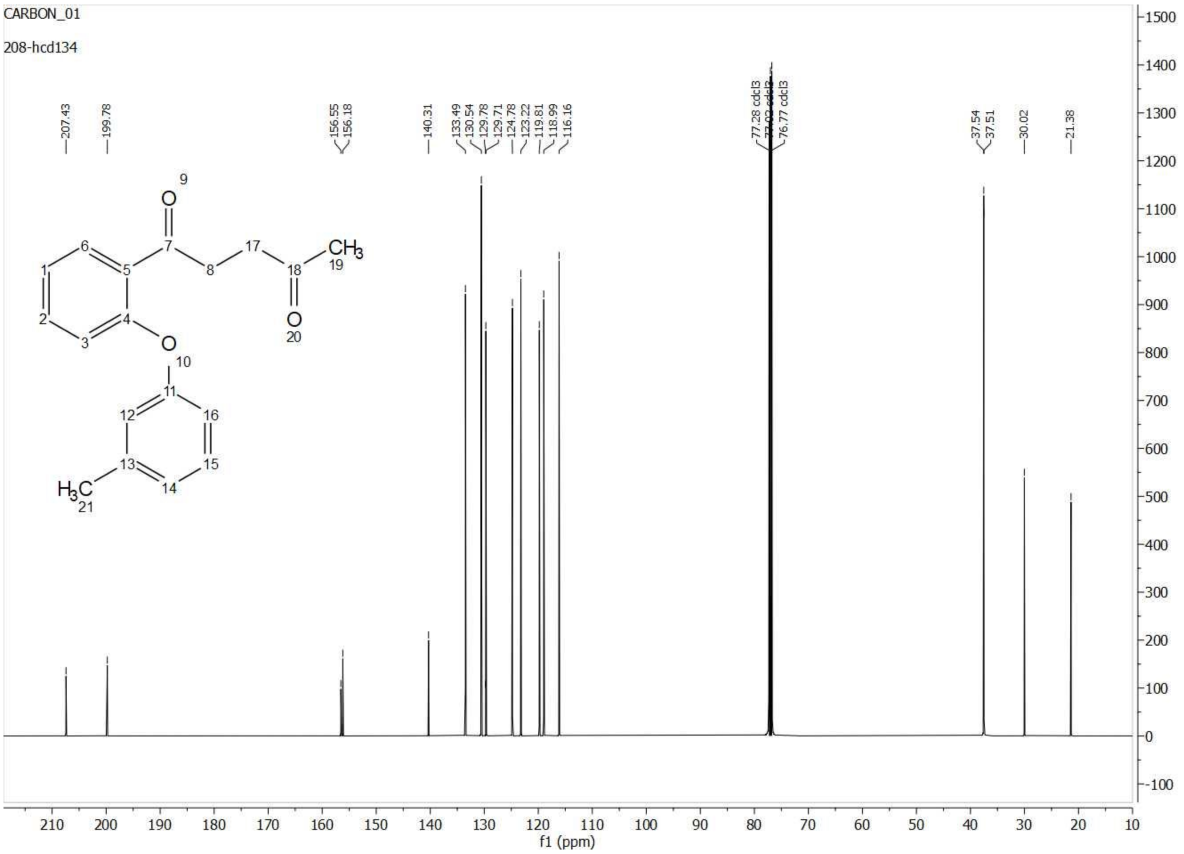

Scheme S23. ^{13}C NMR spectrum of **2i**.

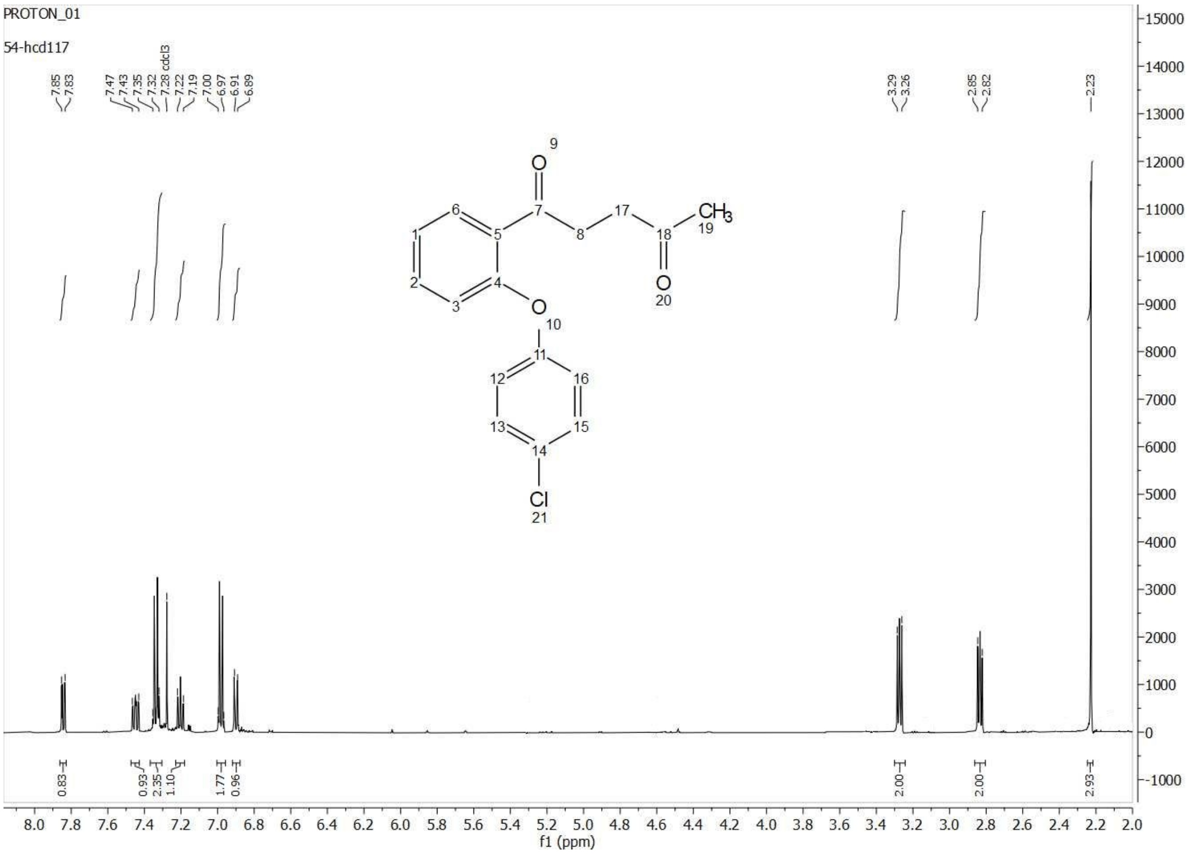

Scheme S24. ^{1}H NMR spectrum of **2j**.

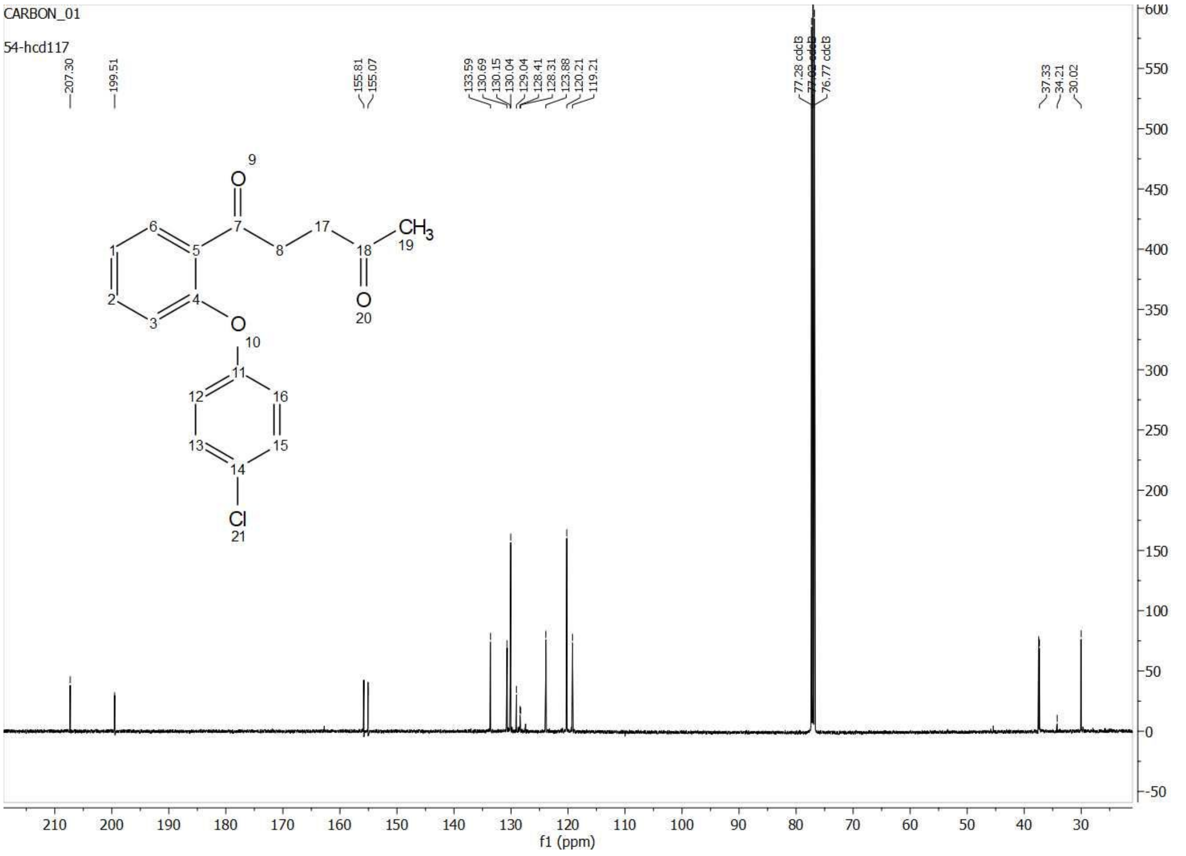

Scheme S25. ^{13}C NMR spectrum of **2j**.

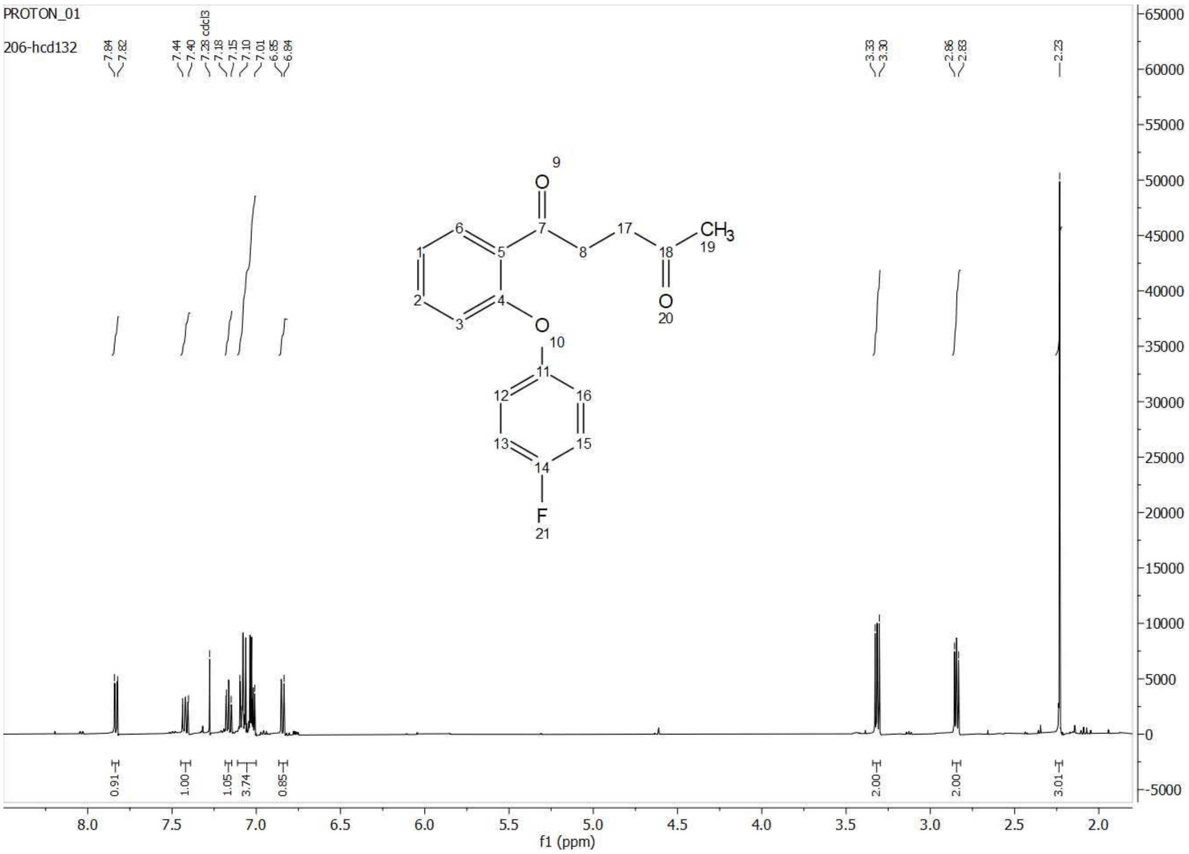

Scheme S26. ^{1}H NMR spectrum of **2k**.

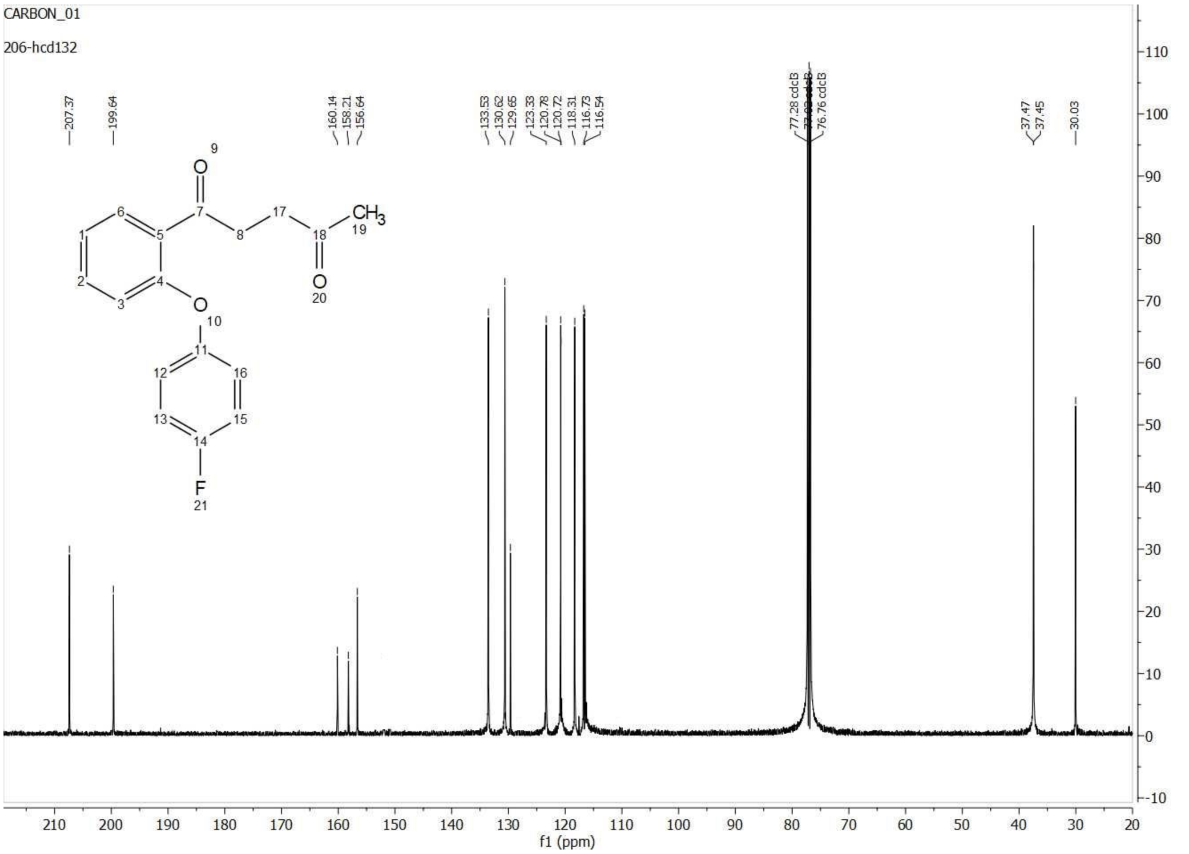

Scheme S27. ^{13}C NMR spectrum of **2k**.

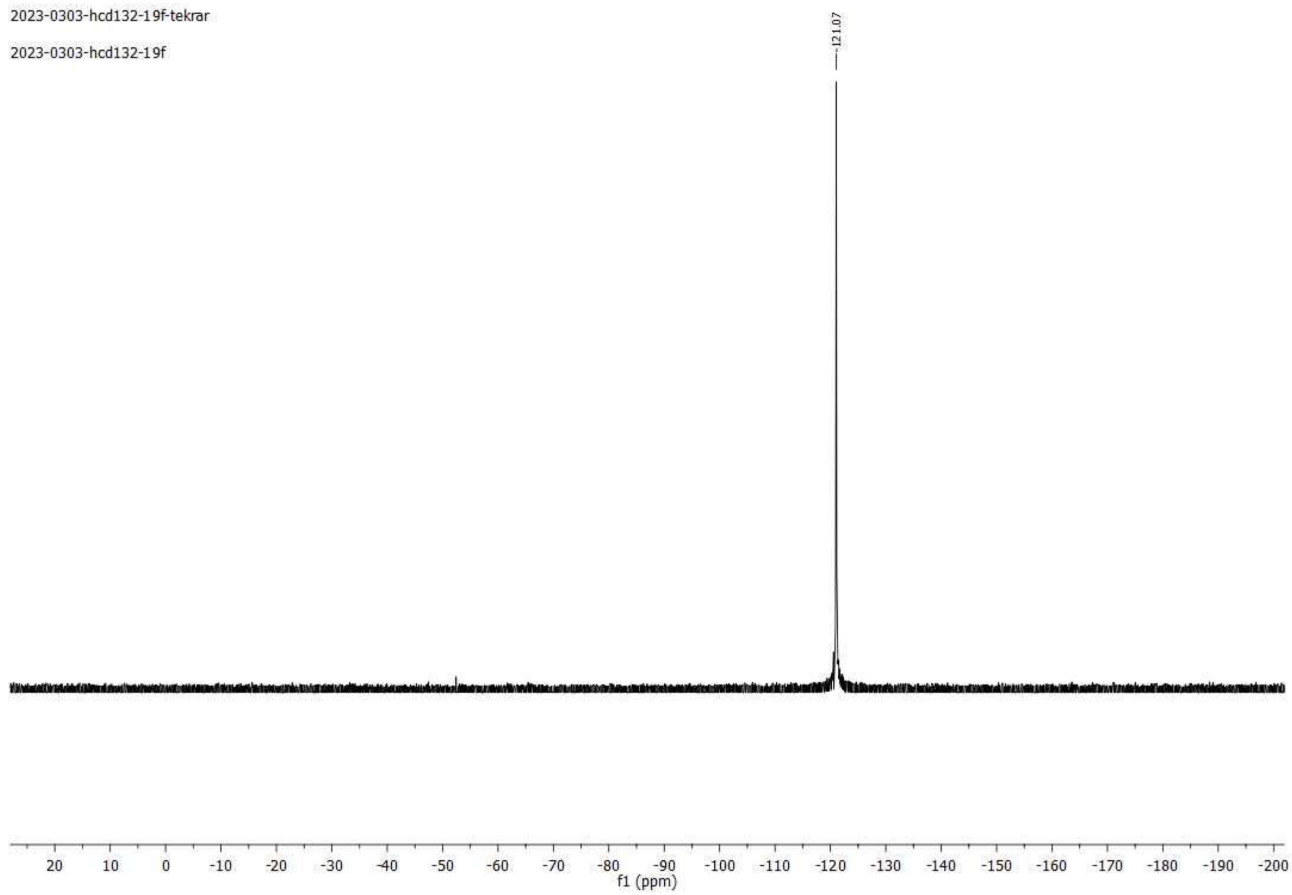

Scheme S28. ^{19}F NMR spectrum of **2k**.

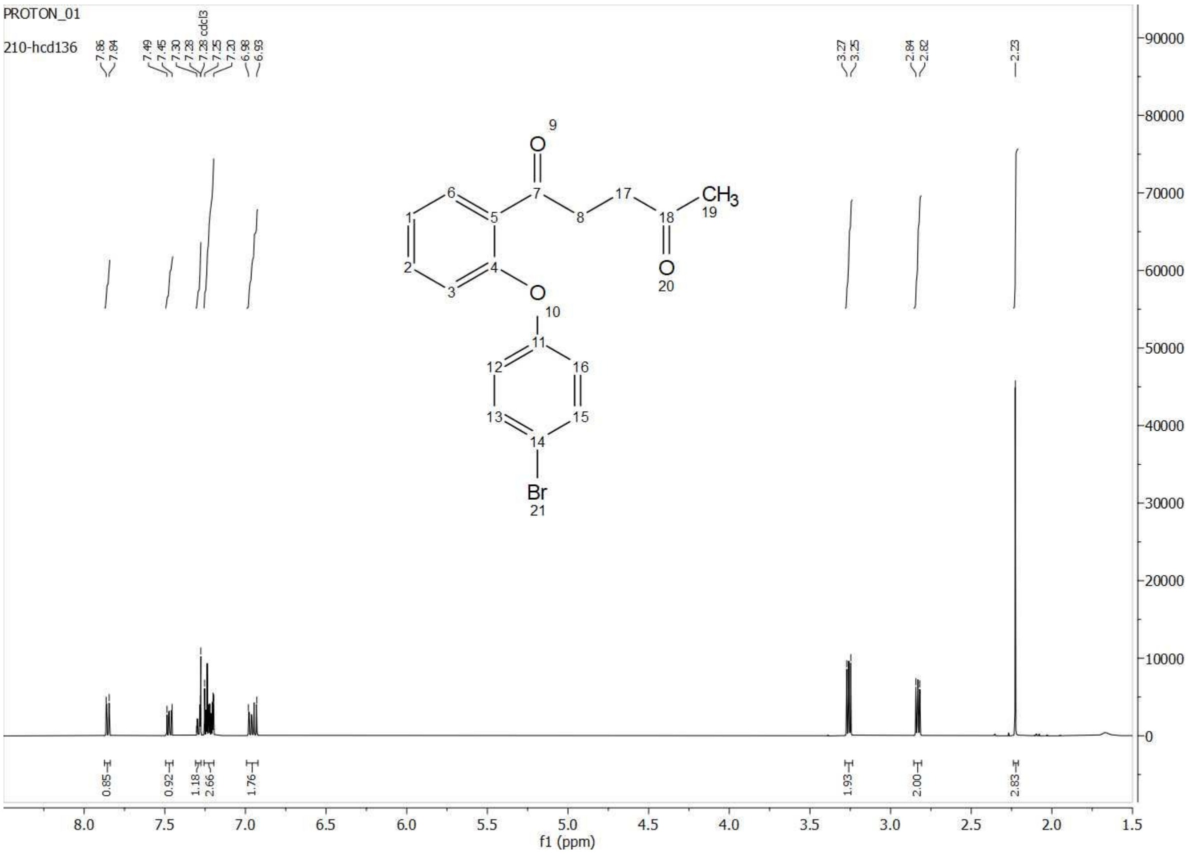

Scheme S29. ^{1}H NMR spectrum of **2l**.

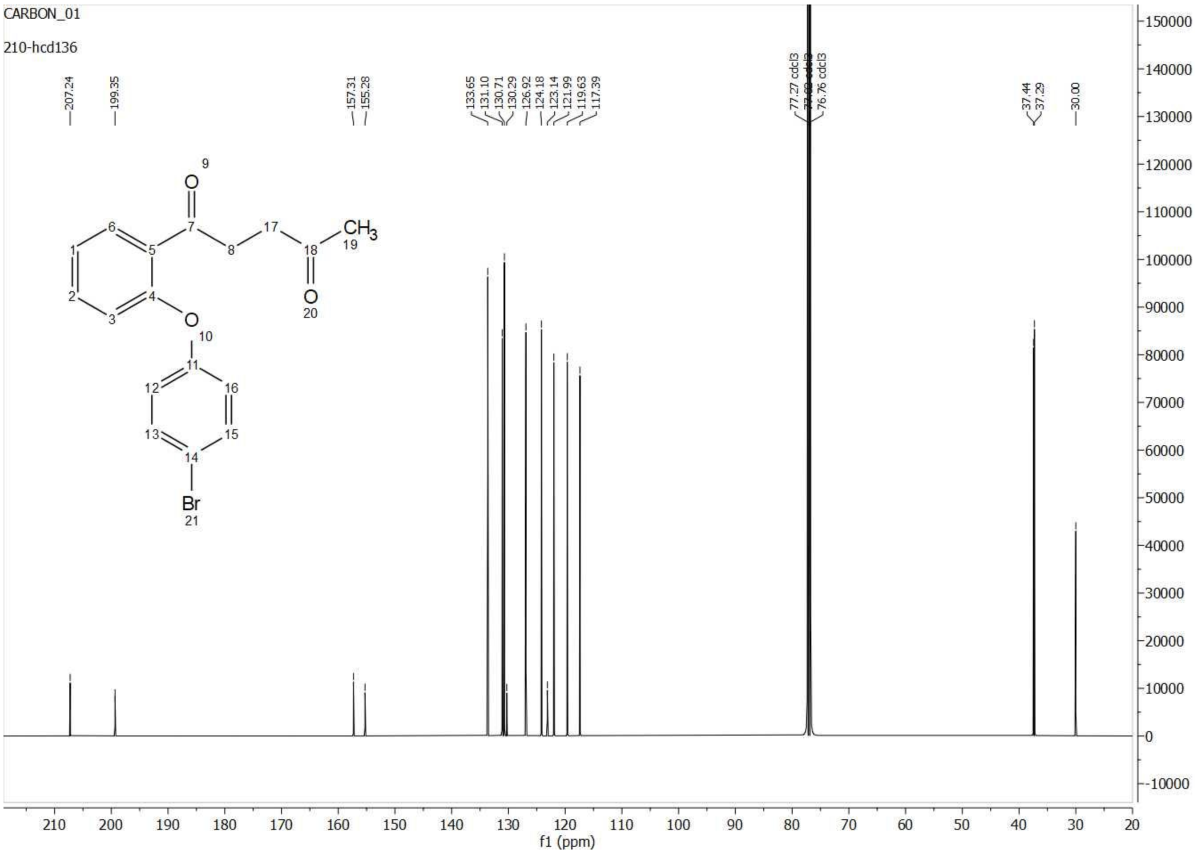

Scheme S30. ^{13}C NMR Spectra of **2l**.

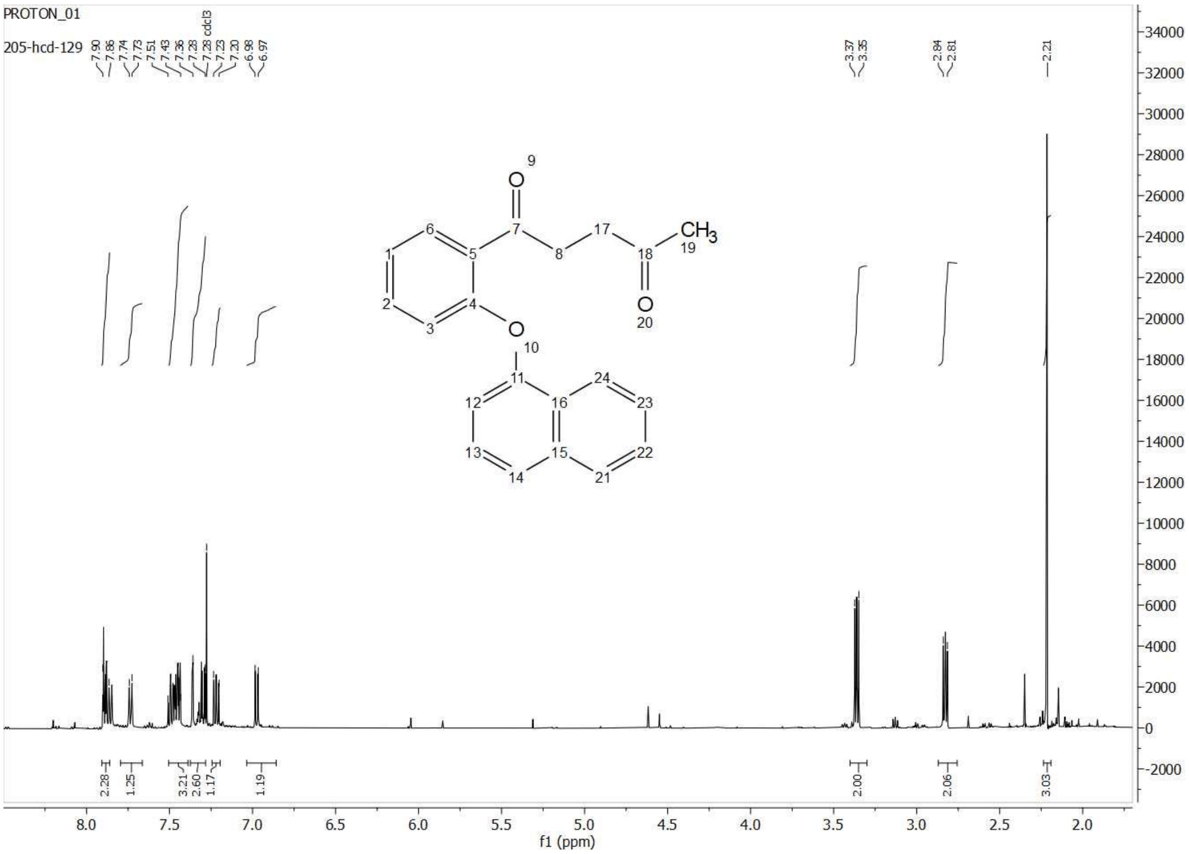

Scheme S31. ^{1}H NMR spectrum of **2m**.

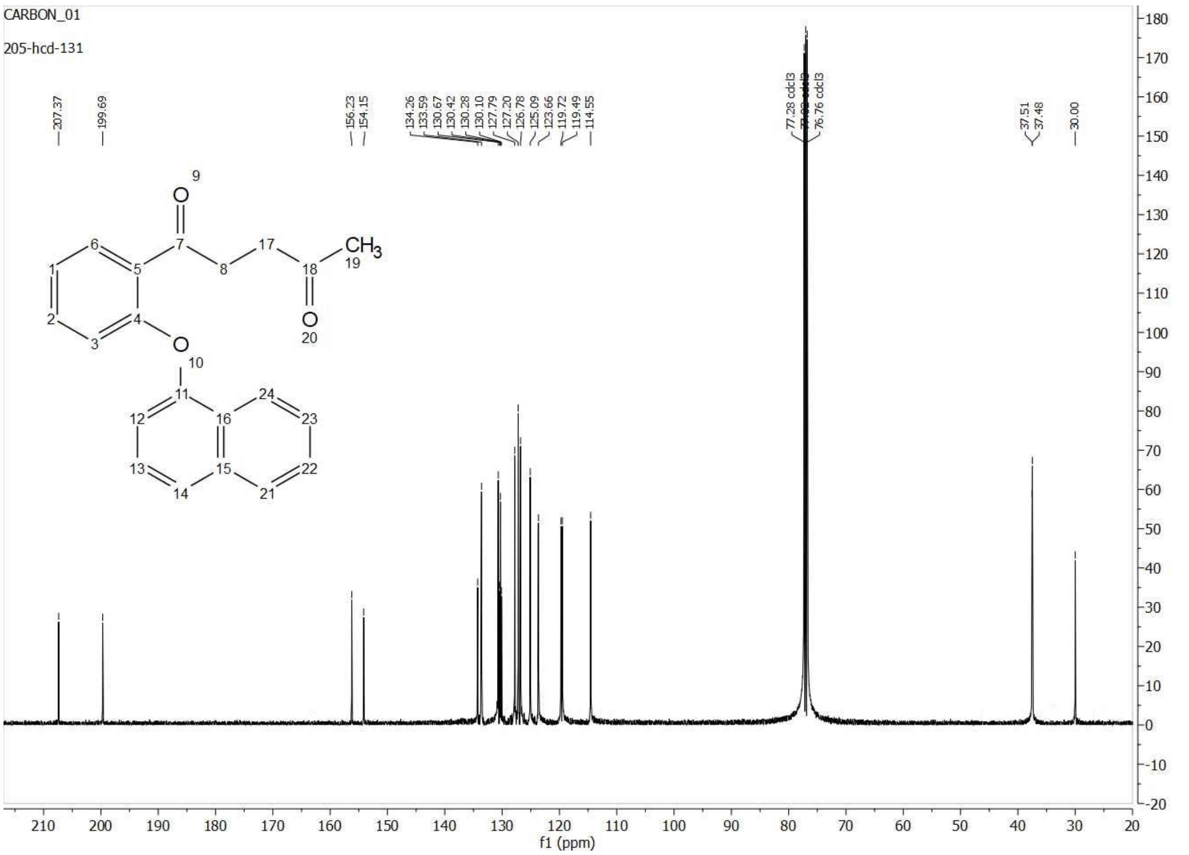

Scheme S32. ^{13}C NMR Spectra of **2m**.

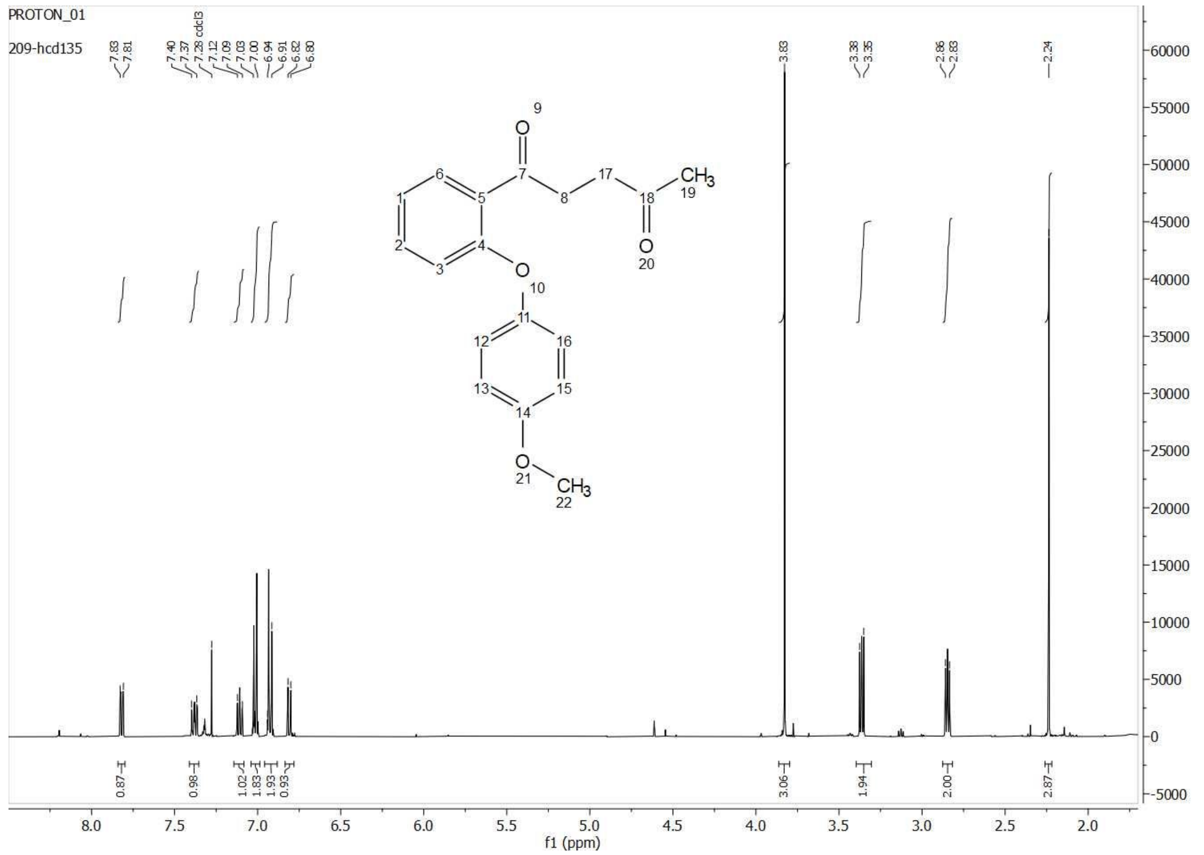

Scheme S33. ^{1}H NMR spectrum of **2n**.

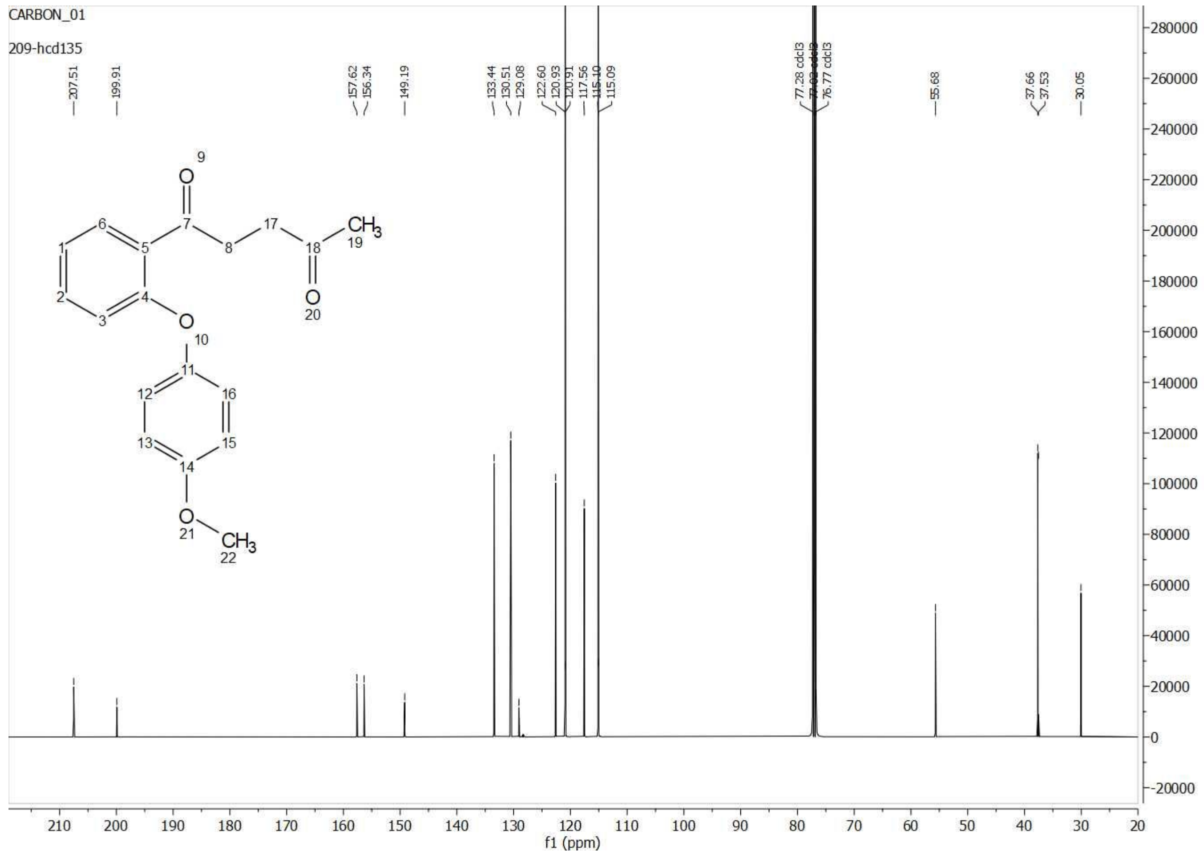

Scheme S34. ^{13}C NMR Spectra of **2n**.

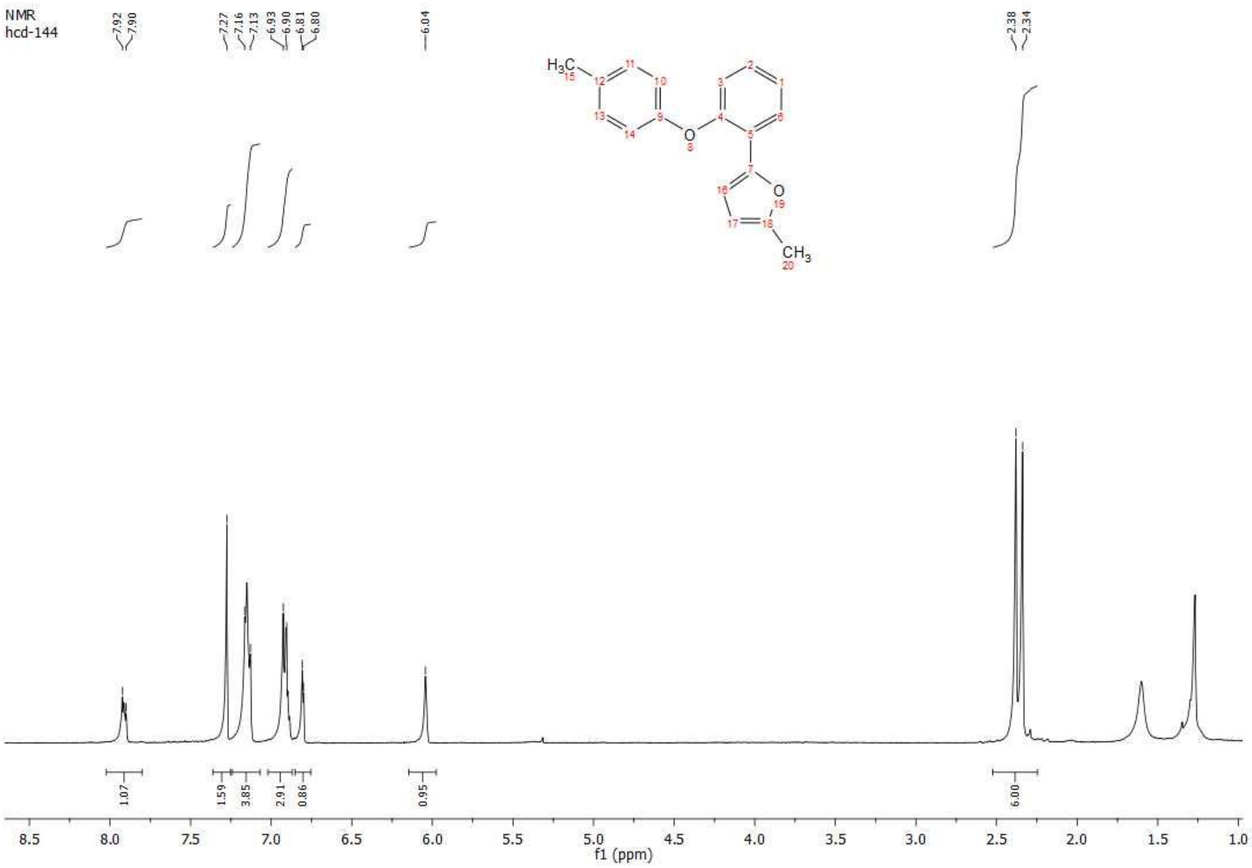

Scheme S35. ^{1}H NMR spectrum of **4a**.

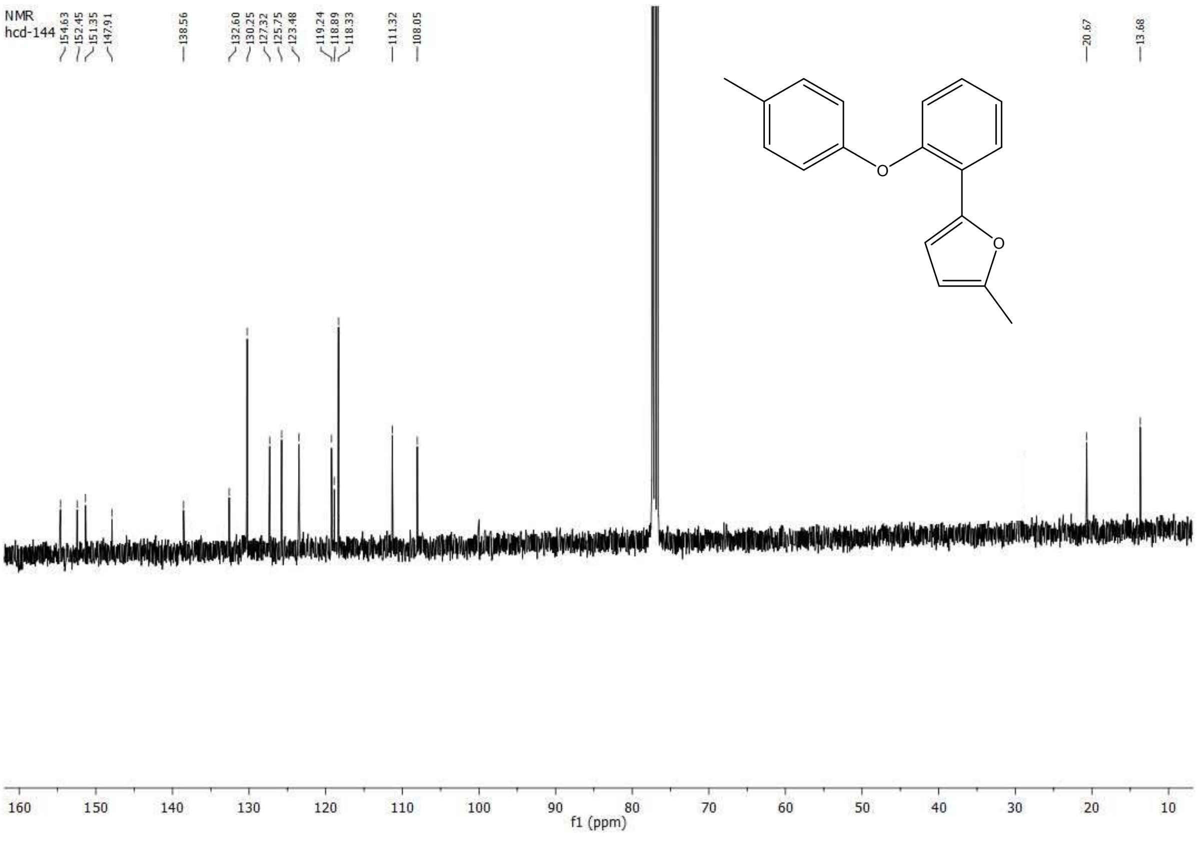

Scheme S36. ^{13}C NMR Spectra of **4a**.

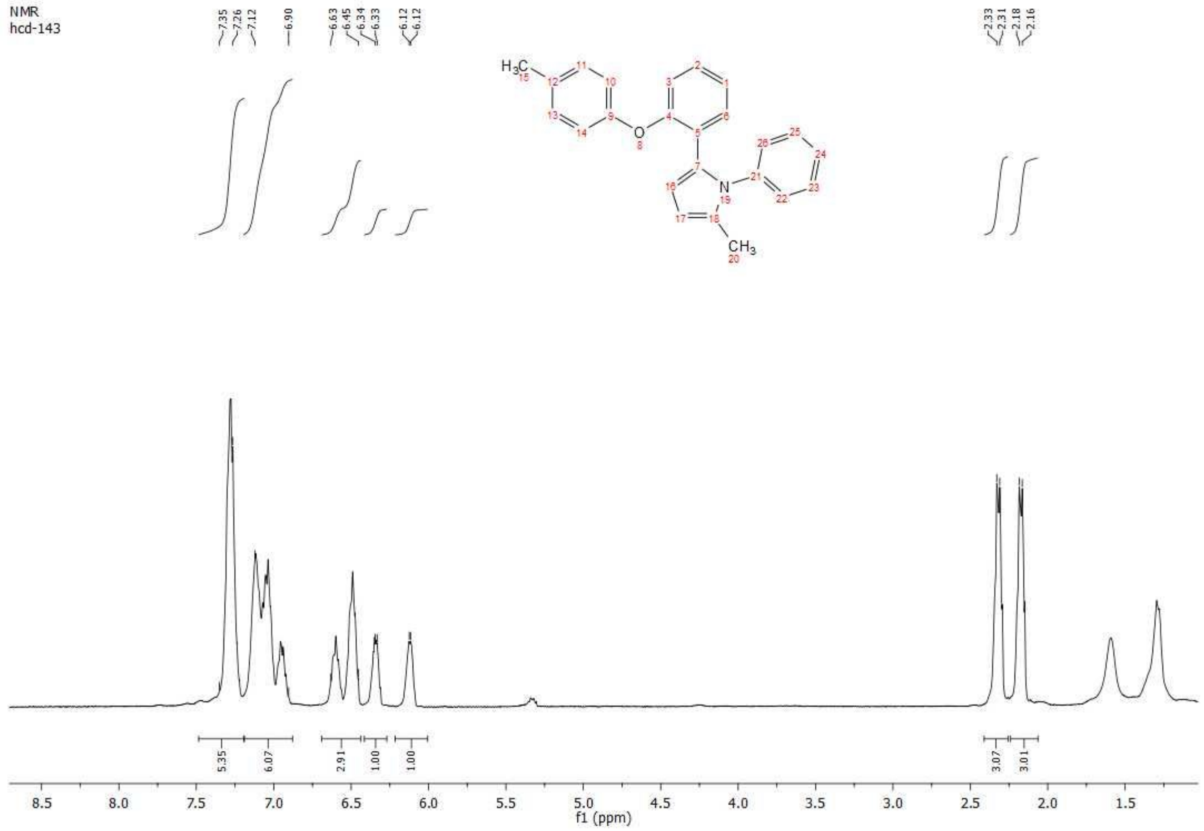

Scheme S37. ^{1}H NMR spectrum of **4b**.

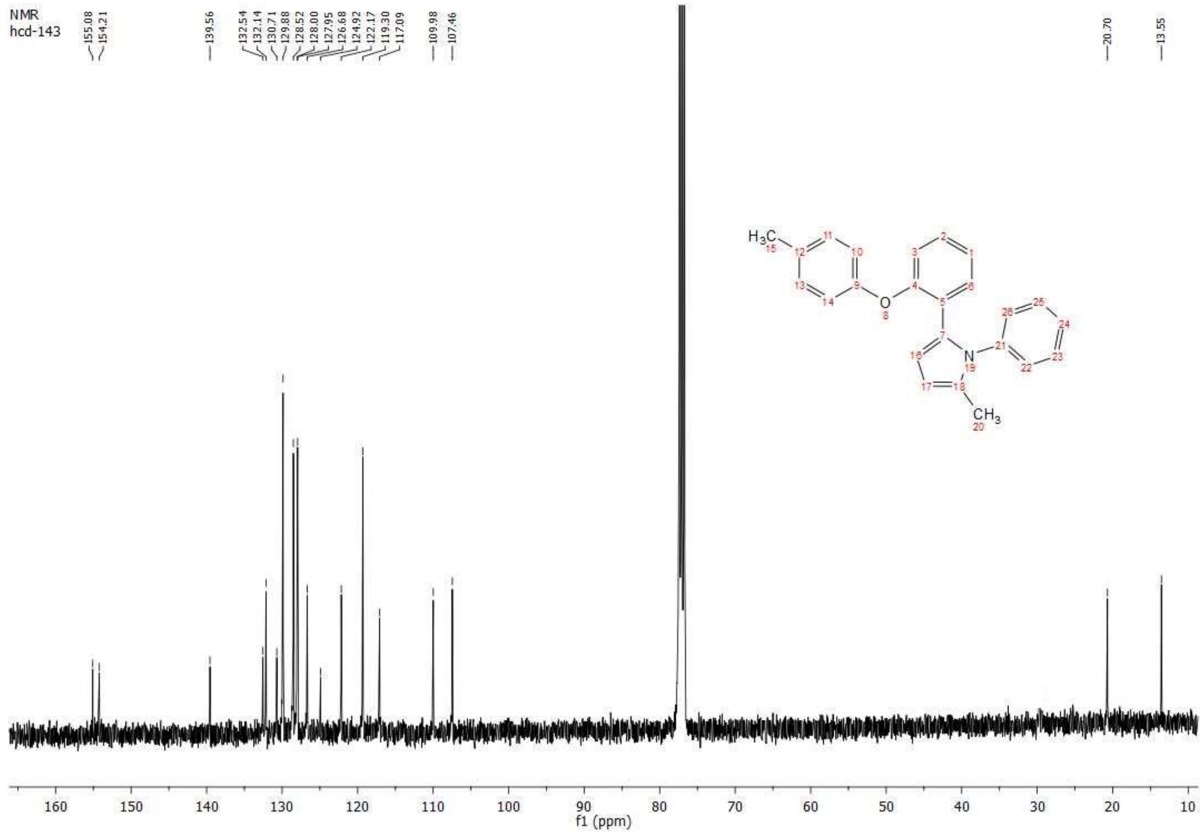

Scheme S38. ^{13}C NMR Spectra of **4b**.

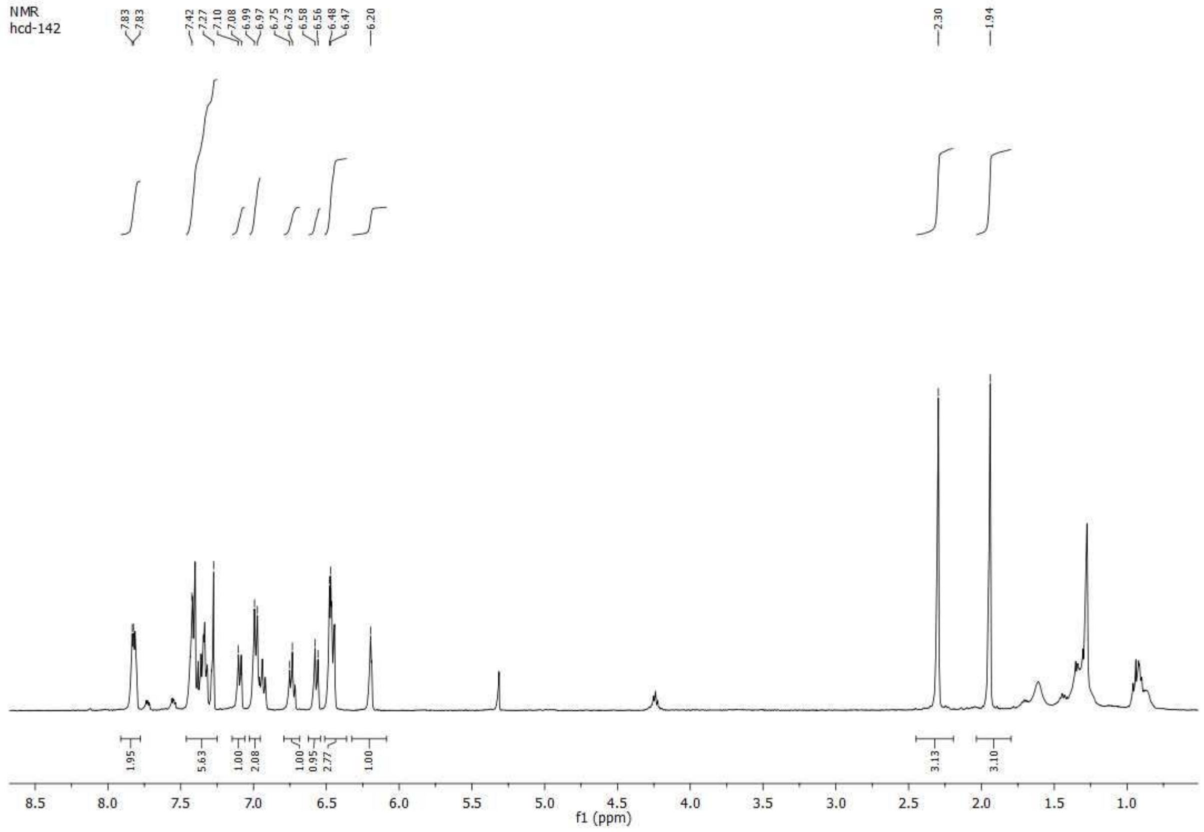

Scheme S39. ^{1}H NMR spectrum of **4c**.

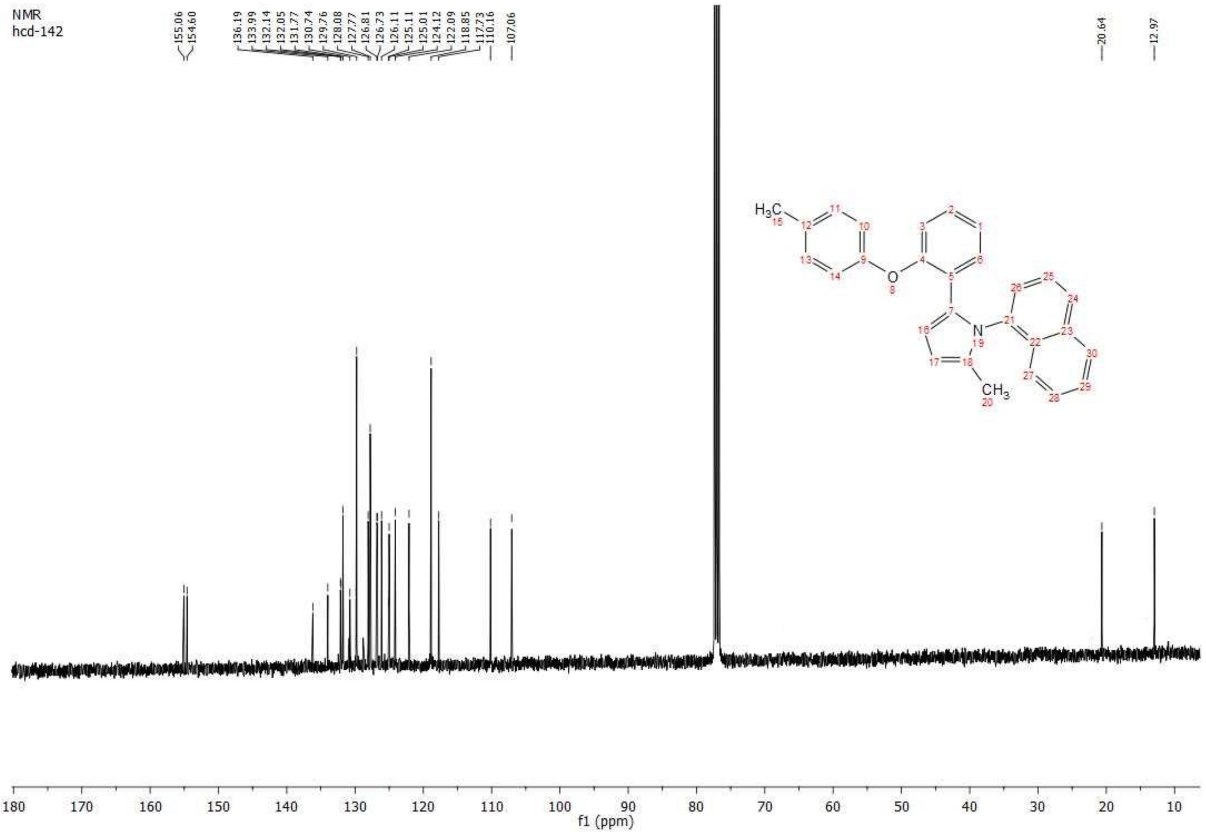

Scheme S40. ^{13}C NMR Spectra of **4c**.
